# Lung disease in relation to unique monocyte-macrophage subpopulations induced by combined inhalant endotoxin and collagen-induced arthritis

**DOI:** 10.3389/fimmu.2025.1557583

**Published:** 2025-04-09

**Authors:** Jill A. Poole, Aaron Schwab, Geoffrey M. Thiele, Todd A. Wyatt, Amy J. Nelson, Oliver W. Schanze, Angela Gleason, Michael J. Duryee, Bryant R. England, Ted R. Mikuls

**Affiliations:** ^1^ Department of Internal Medicine, College of Medicine, Omaha, NE, United States; ^2^ Research Service, Veterans Affairs Nebraska-Western Iowa Health Care System, Omaha, NE, United States; ^3^ Department of Environmental, Agricultural and Occupational Health, College of Public Health, University of Nebraska Medical Center, Omaha, NE, United States

**Keywords:** lung, autoimmunity, macrophage, monocyte, inflammation, fibrosis, transcriptome, imaging

## Abstract

**Background:**

Lung disease is the most overrepresented cause of death in rheumatoid arthritis (RA). Animal studies have demonstrated potentiated autoimmunity, arthritis, and profibrotic/inflammatory lung disease with a combination of airborne exposures and collagen-induced arthritis (CIA), a model that recapitulates features of RA-associated interstitial lung disease (RA-ILD). As patients with RA-ILD demonstrate unique circulating monocyte subpopulations, this study aims to characterize lung infiltrating monocytes/macrophages in a mouse model of RA-ILD and determine whether reducing these cells mitigates the development of lung disease.

**Methods:**

Autoimmune-prone DBA/1J mice received intranasal inhalation of lipopolysaccharide (LPS) daily for up to 5 weeks and CIA induction. Experimental groups included Sham (saline injection/saline inhalation), CIA (CIA/saline), LPS (saline/LPS), and CIA+LPS (CIA/LPS). Lung disease was assessed by longitudinal imaging, lung function measurements, bronchoalveolar lavage fluid, lung tissues, and lung histopathology. Cell subpopulations were analyzed by single cell RNA-sequencing and flow cytometry. Intravenous clodronate liposome administration was employed to reduce circulating monocytes.

**Results:**

Longitudinal imaging demonstrated increased lung volume and tissue density in CIA+LPS mice. Lung function assessment showed reduced compliance and increased airway resistance with dual exposure. Unsupervised clustering revealed 16 discrete clusters among the experimental groups with robust clusters of monocytes/macrophages of overlapping characteristics for both CIA+LPS and CIA. By flow cytometry, dual CIA+LPS exposure induced activated CD11c^+^CD206^+^CD11b^+^MHC Class II^hi^CD80^+^ alveolar macrophages, CD11c^mid^CD206^-^CD11b^+^Ly6C^hi^(and Ly6C^lo^)MHC Class II^hi^CD80^+^CD86^+^ interstitial macrophages, and CD11c^-^CD11b^+^Ly6C^hi^MHC Class II^hi^CD80^+^CD86^+^ monocytic-like cells. MHC Class II^hi^-expressing cells across monocyte/macrophage subpopulations of CIA+LPS treated mice were more aligned with CIA than LPS alone. Intravenous clodronate liposome administration reduced CIA+LPS-induced both CD11c^+^CD11b^+^ and CD11c^mid^CD11b^+^ lung macrophages, neutrophils, lymphocytes, inflammatory/pro-fibrotic mediators, and expression of vimentin and citrullinated and malondialdehyde acetaldehyde (MAA)-modified proteins/lung autoantigens.

**Conclusion:**

The interaction of inhalation-induced airway inflammation and autoimmune arthritis results in lung disease associated with uniquely activated infiltrating inflammatory interstitial macrophages. Moreover, depletion of circulating monocytes attenuated lung disease. Whereas the induced interstitial macrophage immunophenotype is more aligned to CIA than endotoxin exposure, co-exposure modeling renders unique features that potentially inform the pathogenesis and treatment of RA-ILD.

## Introduction

Rheumatoid arthritis (RA) is a debilitating autoimmune disease that is frequently associated with a number of extra-articular manifestations including interstitial lung disease (ILD) (1). Whereas subclinical lung disease affects up to 40% of individuals with RA, clinically relevant ILD impacts up to 10% of patients and respiratory-related diseases are among the leading causes of death in men and women with RA (2–4). The pathogenesis of RA-associated ILD (RA-ILD) is complex with roles for autoimmunity, dysregulation of inflammatory and fibrotic pathways, oxidative stress, and exposures to (5) various environmental and occupational factors in genetically susceptible persons (2, 6, 7). Several inhalant environmental and occupational factors are associated with RA and RA-related lung disease including tobacco smoke, air pollutants, military-related exposures, agriculture, textile, silica, construction, and more (7). As endotoxin is an important component of many of these exposures including agriculture, textile, tobacco smoke, silica, military-related exposures and air pollutants (8–13), it is often utilized to model environmental/occupational inhalant exposures. Interestingly, the link between occupational and other environmental exposures and RA risk is well established in men but less clear in women (14). Whereas cellular mediators of RA-ILD have been postulated to be neutrophils, macrophages, and lymphocytes (15), recent studies have identified increasing myeloid cell subpopulations (particularly monocytes/macrophages) as playing a central pathogenic role in disease development (16–18).

High peripheral blood monocyte counts have been associated with poor prognosis in RA-ILD (16), and we recently found increases in circulating intermediate and nonclassical monocytes (as opposed to classical monocytes) as well as increased monocytic myeloid derived suppressor cells in RA-ILD patients (17). Moreover, circulating monocytes in individuals with RA-ILD demonstrate unique innate immune and fibrotic gene expression profiles compared to patients with idiopathic pulmonary fibrosis, patients with RA without lung disease, and those without autoimmunity and lung disease (18). Although it is likely that these alternatively activated and/or suppressed circulating monocytes ultimately impact lung disease, their potential role in mediating RA-ILD remains unclear. Animal modeling of RA-ILD represents an important strategy to understand disease mechanisms and explore potential interventional strategies. We have previously combined repetitive inhalant airborne hazard exposures (e.g., endotoxin, agriculture organic dust extracts) with collagen-induced arthritis (CIA) in mice as a model of RA-ILD. This model demonstrates several features resembling human RA-ILD including potentiated autoimmunity, arthritis, expression of post-translationally modified lung autoantigens (i.e., citrullinated and malondialdehyde acetaldehyde (MAA)-modified proteins), and pro-fibrotic and pro-inflammatory lung disease characterized by increased lung infiltrates comprised of macrophages, lymphocytes, and neutrophils (19–22).

With the development of newer technologies, new dimensions of the richness and heterogeneity of lung mononuclear phagocytes, including resident and recruited monocytes and macrophages that can adapt to the lung microenvironment to contribute to lung homeostasis, inflammatory stimuli, scavenging, fibrosis processes and immune surveillance exist (23–28). Although tissue-resident macrophages such as alveolar macrophages (AM) may be seeded during embryonic development and maintained in the absence of monocyte recruitment (29), it has also been suggested that circulating monocytes participate in the response to stimuli and the renewal of both AM and interstitial macrophages (IM) as well as persist in the lung as monocytic-like cells (26, 27, 30). The immunophenotype of combined airborne hazard exposure with arthritis induction (CIA)-induced lung monocyte-macrophage subpopulations has not been detailed nor has their role been known, which is the aim of this current study.

The objectives of this study were to leverage the combination of inhalant endotoxin (lipopolysaccharide, LPS) and CIA (CIA+LPS) to further characterize the lung pathology and lung function consequences of this model relative to LPS alone, CIA alone, and control mice and apply single-cell RNA sequencing and unsupervised clustering to resultant lung immune cells. Next, lung monocyte-macrophage subpopulations were delineated across groups followed by studies examining the impact of selective depletion of circulating monocytes to better understand the contribution of these cells to RA-ILD development.

## Methods

### Animal exposure model

An established 5-week exposure model (19, 20) was utilized whereby mice were randomized to 1 of 4 experimental groups including Sham (saline injection/saline inhalation), LPS alone (saline injection/LPS inhalation), collagen-induced arthritis (CIA) alone (CIA induction/saline inhalation), and CIA+LPS co-exposure (CIA injection/LPS inhalation). DBA/1J mice (age 6-8 weeks) were purchased from Inovtiv (Indianapolis, IN) and allowed to acclimate for one week prior to initiation of experiments. Male mice were utilized for all studies because we have previously demonstrated that female mice were profoundly less susceptible to development of lung disease in the setting of arthritis induction (19). CIA was induced as per the Chondrex protocol (Chondrex, Inc, Redmond, WA) (22). Airway inflammation was induced using an established model of repetitive intranasal LPS inhalation (19). Under light sedation with isoflurane, mice received 100 ng of LPS from gram-negative *Escherichia coli* (O55:B5; Sigma, St. Louis, MO) in 50 μl of sterile saline or saline alone daily for up to 5 weeks (weekends excluded). Animals were euthanized 1 day after the final LPS exposure by isoflurane followed by exsanguination (right axillary blood collection). No respiratory distress, signs of stress, or significant weight loss (defined as >20%) were observed throughout the exposure period.

All animal studies are detailed in accordance with the Animal Research Reporting *In Vivo* Experiments (ARRIVE) guidelines (https://arriveguidelines.org/). Animal procedures were approved by the Institutional Animal Care and Use Committee and were in accordance with the NIH guidelines for the use of rodents.

### Clodronate-induced systemic monocyte/macrophage depletion

In separate proof-of-principle studies, Sham and CIA+LPS co-exposed mice were administered encapsulated clodronate liposomes intravenously to deplete systemic monocytes and recruited monocyte-derived macrophages (31, 32). Prior to CIA injections and LPS inhalation exposures, control and clodronate liposomes (Liposoma Technology, Amsterdam, Netherlands; 200 μl x 5 mg/ml) were injected weekly x 2 doses into the tail vein. The timing used was based on the reported duration of effect with repletion of macrophages observed 1-2 weeks following clodronate administration (33). In these studies, co-exposure duration was limited to 2 weeks due to increased tail vein irritation/inflammation noted with weekly liposome injections plus CIA at ≥ 3 weeks.

### Micro-CT imaging

Progression of lung pathology was longitudinally and quantitatively determined using a live animal Quantum GX-2 micro-CT scanner (Perkin Elmer, Waltham, MA) with measurements at baseline and weekly for 5 weeks of treatment conditions. High-speed, 4-minute scans were performed utilizing an X-ray tube voltage of 90 kV and a current of 88 μA as previously described (34). Model construction and quantification were completed using 3D Slicer (5.6.2) software. Registration between baseline and subsequent scans was performed using the Elastix extension (35). Fibrosis progression is associated with increased lung volumes (i.e., increase in non-aerated lung volume) (36–39) and increased Hounsfield Units (HU) density distribution (i.e., increased tissue density) (40, 41).

### Invasive pulmonary function measurements

Lung function was assessed for dynamic compliance (Cdyn) and total lung resistance (R_L_) was invasively assessed via a tracheostomy tube at 5 weeks following treatment conditions using a computerized small-animal ventilator (FinePointe, Buxco Electronics, Wilmington, NC) as previously described (42).

### Arthritis evaluation

A semi-quantitative arthritis scoring system (Chondrex; www.chondrex.com) was applied weekly and based on hind-foot examination with score ranges from 0 (no inflammation) to 4 (erythema and severe swelling encompassing ankle, foot, and digits).

### Single-cell RNA sequencing

For these studies, 4-5 mice per treatment group were euthanized with whole lungs harvested after removal of blood from the pulmonary vasculature. Lungs were dissociated with gentleMACS Dissociator (Miltenyi Biotech, Gaithersburg, MD) in a digestion solution as previously described (43). Sample were then pooled by treatment group. Single cell suspensions processed for RNAseq generated from whole lungs were quantified and viability tested using a LUNA-FL™ Dual Fluorescence Cell Counter (Logos Biosystems, Annandale, VA). Single cells were isolated using a 10x Chromium controller per manufacturer’s suggested protocol (10x Genomics, Pleasanton, CA). Reverse transcription was performed on a thermocycler (C100 Touch™ Thermal cycler, Bio-Rad, Hercules, CA) per recommended protocol followed by cDNA amplification. Amplified products were solid phase reversible immobilization (SPIR) bead-purified and evaluated by Fragment Analyzer (Agilent, Santa Clara, CA). Twenty-five percent of the cDNA volume was subjected to fragmentation and double-sided SPRIselect (Beckman Coulter, Indianapolis, IN) was used for PCR purification. PCR products were purified, quantified, and library size distribution was determined by fragment Analyzer. Libraries were sequenced per the manufacturer’s suggested parameters on a NextSeq500 and sequenced to an average depth of 50,000 reads per cell.

### Single-cell RNA sequencing data processing

Cell Ranger Count tool uses STAR to align the reads to the mouse reference transcriptome. Aligned reads were filtered for valid cell barcodes and unique molecular identifiers (UMIs) and observed cell barcodes were retained. The default estimated cell count was used for this experiment. The resulting analysis files for each sample were then aggregated using the Cell Ranger aggr pipeline, which performed a between-sample normalization step and merged all samples into one.

The aggregated cell-by-gene count matrix was then used as the input for downstream analysis. The aggregated matrix was analyzed using the Loupe Browser (v.6.4; 10X Genomics) that allows easy visualization and analysis of Chromium single-cell 5′ and 3′ gene expression data and visualize UMAP plots, as well as normalized and annotated cell clustering and projection. To find the Differential Expression or Marker genes, we used the “globally distinguished” from the Significant Feature Comparison option to distinguish a cluster from every other cluster by using the p-value threshold (0.05) and log_2_ fold-change (FC) > 1 (upregulated) or < -1 (downregulated) to filter for significant genes. The data sets are deposited to the Gene Expression Omnibus (GEO) database with access number GSE284234.

### Flow cytometry

Total lung cells (bronchoalveolar lavage fluid remained intact) were quantified following tissue dissociation of right lung lobes (19). Single-cell suspensions were stained with LIVE/DEAD Fixable Dead Cell stain (Invitrogen, Carlsbad, CA) and incubated with CD16/32 (Fc Block, Biolegend, San Diego, CA) to minimize non-specific antibody staining. Cells were then stained with antibodies (BD Biosciences, Franklin Lakes, NJ unless otherwise stated) against mouse cell surface markers including rat anti-mouse CD45 (clone:30-F11), CD11b (M1/70), Ly6G (1A8), CD86 (GL-1), Ly6C (HK1.4), CD206 (C068C2), F4/80 (T45-2342), CD4 (RM4-5), C8a (53-6.7), CD19 (1D3, Invitrogen), MHC Class II (I-A/I-E) (M5/114.15.2, Cell Signaling Technology), hamster anti-mouse CD3e (145-2C11), Armenian Hamster anti-Mouse CD11c (N418, Invitrogen), or Armenian Hamster anti-Mouse CD80 (16-10A1). Cells were acquired on a BD LSRII Yellow/Green cytometer configured with 355-nm, 405-nm, 488-nm, 561-nm, 633-nm lasers. Post-acquisition, data were exported and stored using the flow cytometry standard (FCS) 3.1 format and analyzed using FlowJo software version 10.10 (FlowJo, Ashland, OR).

The gating strategy for identification of non-debris, singlets, live CD45^+^ cells is depicted in **Supplementary Figure 1**. Lung immune cells were gated and defined as neutrophils (CD11c^-^Ly6G^+^), CD3^+^CD4^+^ T cells, CD3^+^CD8^+^ T cells and CD19^+^ B cells, as previously reported (19, 31, 44). Monocytic and macrophage populations were first broadly categorized as alveolar macrophages (AM) (CD11c^+^CD206^+^CD11b^var^), interstitial macrophages (IM) (CD11c^mid^CD206^-^CD11b^+^), and monocytic-like cells (CD11c^-^CD206^-^CD11b^+^), based upon reports by others (45–47). Note, Fluorescence Minus One (FMO) controls were used to set the upper boundary for background signal for monocyte/macrophage subpopulation expression of CD206, MHC Class II, Ly6C, CD80, CD86 (**Supplementary Figure 2**). To further delineate subpopulations utilizing reports by others (45–47), “resting” AMs were defined as CD11c^+^CD206^+^CD11b^-^CD80^+^MHC Class II^lo^ (47). As recent reports by others have defined lung CD11c^+(hi)^CD206^+^CD80^+^ macrophages that highly express CD11b and/or MHC Class II as either activated AM or interstitial macrophages (IM) (27, 31, 45–48), in this current study, CD11c^+^CD206^+^CD80^+^MHC Class II^hi^CD11b^-^ were referred to as alternatively activated AMs and CD11c^+^CD206^+^CD80^+^MHC Class II^hi^CD11b^+^ as activated AMs. IMs (CD11c^mid^CD206^-^CD11b^+^) and monocytic-like cells (CD11c^lo^CD206^-^CD11b^+^) were delineated into descriptive subpopulations based upon MHC Class II and Ly6C expression with corresponding co-stimulatory molecule (CD80, CD86) expression by subpopulation. Ly6C is a glycoprotein utilized to identify lung macrophage and monocyte subpopulations in a steady state vs. activated/disease state with high Ly6C expression associated with inflammatory and “recruited” monocyte and macrophage status with conversion to low expression with resolution of disease processes (27). Moreover, Ly6C^hi^-expressing lung monocytes/macrophages has been associated with pro-fibrotic features (49). Correspondingly, MHC Class II high expression is characteristically associated with a “M1”-activated/inflammatory-like phenotype (27, 45–48). Lung cell population numbers were determined by multiplying percent gated by total lung cells.

### Clustering T-distributed stochastic neighbor embedding

We utilized t-distributed stochastic neighbor embedding (tSNE) as an unsupervised nonlinear dimensionality reduction algorithm to visualize the high dimensional flow cytometry data generated (50). The FlowJo tSNE platform computes clustering of data from user defined selection of cytometric parameters (50). To reduce noise, non-debris singlet, live CD45^+^, Ly6G^-^, lymphocyte gate^-^ events from a representative sample from each experimental group at 30,000 events/sample were concatenated into a single file and analyzed by tSNE with selection of cytometric parameters including FSC-A, SSC-A, and uncompensated CD11c, CD11b, Ly6C, MHC Class II, CD86, CD80, CD206, and F4/80 with the following technical options: iterations:1000, perplexity:20, learning rate:14,000, *k-*nearest neighbors (KNN) algorithm: Exact-vantage point tree, and gradient algorithm: Barnes-Hut. After computation, the embedded tSNE mapping of the combined 4 experimental groups is shown by contour plots of tSNE_1 vs. tSNE_2 with overlay labeling based upon the gating strategies. The 4 representative groups were also separated for visualization.

### Phagocytosis

To assay functional consequences associated with experimental conditions, phagocytic ability was determined for lung monocyte/macrophage subpopulations. Following dissociation of the left lung, cells from 2-3 mice were pooled and single cell suspensions were incubated with opsonized, fluorescein-conjugated *E. coli* BioParticles (Invitrogen) to quantify phagocytic activity. Cells were then placed on ice and incubated as described above for markers indicative of monocytes and macrophages (i.e., live/dead, CD45, CD11b, CD11c). Cells were then cold washed, fixed with 4% paraformaldehyde, and analyzed on a BD LSRII YG (Green Profile). CD11c^+^CD11b^var^, CD11c^mid^CD11b^+^, CD11c^-^CD11b^+^ cells were analyzed for BioParticle-associated fluorescence (44) with results presented as the percent of the cell population exhibiting Bioparticles (% Bioparticle +). To compare phagocytosis across experiments (2 independent experimental runs, n=10 mice/group total with 4 samples/group), the % Bioparticle + per mouse was divided by mean for same specific cell subpopulation in Sham mice to generate a phagocytic index.

### Lavage fluid cells and lung homogenate analyses

Bronchoalveolar lavage fluid (BALF) was collected using 3 x 1 mL of phosphate-buffered saline (PBS; pH 7.2). Total BALF cell counts from pooled lavages were enumerated using a BioRad TC 20 cell counter. Differential cell counts were determined from cytospin-prepared slides (Wescor Cytopro 7620 Cytocentrifuge) with Diff-Quick (Siemens, Newark, DE). Cell-free BALF from the first lavage fraction was evaluated for cytokines and chemokines. After BALF isolation and removal of blood from pulmonary vasculature, right lobe homogenates were prepared (19). BALF and lung homogenates were analyzed for inflammatory/profibrotic mediators by ELISA (R&D Systems, Minneapolis, MN and Abcam, Waltham, MA). Analytes quantified (and thresholds for detection in pg/ml) included tumor necrosis factor (TNF)-⍺ (1.88), IL-6 (1.6), CCL2 (0.304), CCL7 (1.53), CXCL2 (1.5), neutrophil elastase (1.60), fibronectin (0.48 ng/ml), matrix metalloproteinase (MMP)-3 (125), MMP-8 (53), MMP-9 (78.1), and tissue inhibitor of metalloproteinase (TIMP)-1 (31.3).

### Lung histopathology and autoantigens

Left lung lobes were excised and inflated to 15 cm H_2_0 pressure with 10% formalin (Fisher Scientific, Fair Lawn, NJ) for 24 hours to preserve pulmonary architecture (19). Lungs were processed and cut (4-5 μM) at midpoint sections to include regions of both large and small airways as well as blood vessels. Tissues were stained for hematoxylin and eosin (H&E), modified Masson’s Trichrome, or preserved for subsequent immunohistochemistry (IHC) by the Tissue Science Core Facility at the Department of Pathology and Microbiology as previously described (19). H&E-stained slides of the entire lung section from each animal were reviewed and semi-quantitatively scored by an expert pathologist blinded to experimental conditions (19, 51). This scoring system (scored 0 to 4) evaluates the spectrum of inflammatory changes for alveolar and bronchiolar compartments with higher scores indicating greater inflammation (19, 51).

The CC motif chemokine receptor 2 (CCR2) is a critical facilitator of monocyte recruitment and activation that upon reaching inflamed tissue can differentiate into macrophages (52, 53). To quantify CCR2^+^ inflammatory monocytes/macrophages and neutrophils, lung sections were stained with anti-CCR2 (1:100, ab273050 Abcam) and anti-myeloperoxidase (MPO), 1:100 Cat# ab9535, Abcam) and cross absorbed with donkey anti-rabbit AlexaFluor488 (A21206, Lot#2156521, Invitrogen) and AlexFluor555 (Cat#A31572, Invitrogen), respectively, at 1:100 dilution as secondary antibodies. Slides were mounted with VECTASHIELD® Antifade Mounting Medium with 4’,6-diamidino-2-phenylindole; to identify nuclei) (Cat#H-1200, Vector Laboratories, Newark, CA) and visualized under a Zeiss fluorescent microscope (Zeiss Observer.Z1 Zeiss, White Plains, NY). Vimentin is an extracellular matrix protein that is also targeted by post-translation modification generated during the process of inflammation and previously demonstrated to be increased with CIA+LPS co-exposure (19, 20). Citrullinated (CIT) and malondialdehyde acetaldehyde (MAA)-modified proteins are post-translationally modified proteins and lung autoantigens that have also been increased with CIA+LPS co-exposure (19, 20). We have previously demonstrated increased expression and robust co-localization of MAA and CIT with vimentin in the lungs of mice and humans with inflammatory arthritis and lung disease including individuals with RA-ILD (21, 54). Lung sections were stained with Cy5 rabbit anti-vimentin (Bioss, Woburn, MA), Zenon AF594 label (Invitrogen), mouse monoclonal anti-peptidyl-citrulline antibody (clone F95 IgMκ, Millipore Sigma, Burlington, MA) or rabbit polyclonal IgG antibody to MAA (21). Detection of the F95 antibody was done using an AF488 AffiniPure donkey anti-mouse IgM, μ chain specific antibody (Jackson Immunoresearch, West Grove, PA). DAPI was added and samples sealed with Fluoromount-G (Southern Biotech, Birmingham, AL). Fluorochromes were detected using a Revolve fluorescent microscope (ECHO, Sand Diego, CA). For IHC, 10 images of the lung parenchyma per mouse were captured and antigen expression was quantified by integrated density using Image J FIJI plugin (20, 21).

### Statistical analysis

Unless otherwise noted, sample sizes were extrapolated for modulating lung monocyte-macrophage subpopulations from previous work assessing combined CIA+LPS models (19) and LPS administration in the presence/absence of clodronate liposomes (31). The mean (± SD) number of CD11c^int^CD11b^hi^ transition/recruited monocyte-Mϕ was 0.15x10^5^ (0.04 x 10^5^) with Sham and 2.5 x 10^5^ (0.7 x 10^5^) with CIA+LPS co-exposure at 5 weeks; thus, a sample size of n=2 in each group achieved 80% power at the 0.05 level of significance to detect differences in this cell subpopulation following an experimental exposure vs Sham. Likewise, a sample size of n=5 achieved 80% power at the 0.05 level of significance to detect a 60% reduction following targeted cell depletion with clodronate. For micro-CT and lung function studies, there were 5 mice/group. For immunophenotyping of cell infiltrates across 4 groups there were 10 mice in each of the 4 experimental groups. For the clodronate liposome studies using 3 groups (i.e., Sham, vehicle liposome in CIA+LPS, and clodronate liposome in CIA+LPS), there were 8 mice in each group. Sample numbers below the maximum number reflect limitations in available sample quantity.

All data are depicted as mean with standard deviation (SD). The Shapiro-Wilk test was utilized to test for normality among treatment groups. If the assumption of normality was satisfied, a one-way ANOVA was used. For non-parametric data, a Kruskal-Wallis test was applied. The two-stage Benjamini, Krieger, & Yekutieli procedure was applied to control for false discovery (55). All statistical analyses were performed using GraphPad Prism (version: 10.2.2) software, and statistical significance was accepted at a p value < 0.05.

## Results

### Combined CIA+LPS exposure results in increased non-aerated lung volume and tissue density

**Figure 1:**
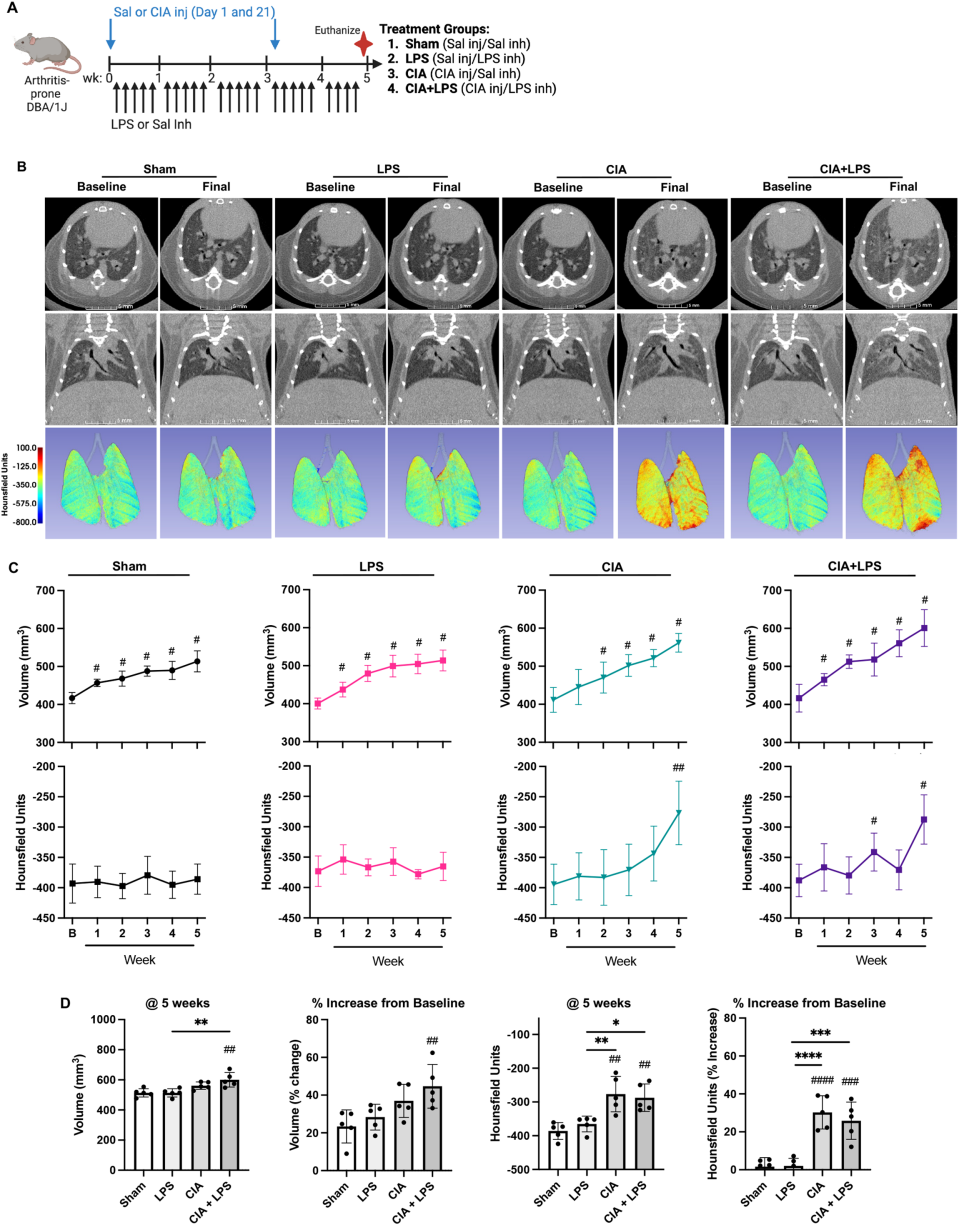
CIA+LPS co-exposure results in increased non-aerated lung volume and tissue density. **(A)** Experimental schematic with mice randomized to receive saline (Sal) or collagen-induced arthritis (CIA) injection (inj) and either daily lipopolysaccharide (LPS) or Sal intranasal inhalation (inh) daily for 5 weeks. **(B)** Representative micro-CT images (axial and coronal plane) at baseline and week 5 (final) from one mouse per treatment group. Density of lung tissues demonstrated by colored range of Hounsfield units (HU). **(C)** Line graphs depict mean volume and averaged HU by treatment group at baseline (B) and over time. **(D)** Scatter plots depict source measurement and % change from baseline of final (5 week) of volume and HU across treatment groups. All data expressed as mean with SD bars of n=5 mice/group. *#* represent statistical significance versus Sham or baseline and brackets with * between groups. ^####,****^<0.0001; ^###,***^0.0001 to 0.001; ^##,**^0.001 to 0.1, ^#,*^0.01 to 0.05.

In the first set of experiments, mice randomized to Sham, LPS, CIA, and CIA+LPS (experimental schematic, **Figure 1A**) underwent live-animal micro-CT imaging to visualize and quantitate lung parenchymal changes including lung volume and density (quantified in Hounsfield Units or HU)(**Figures 1B–D**). Across all treatment groups, quantitative CT measurements demonstrated increases in lung volumes over time as compared to respective baseline measurements (**Figure 1C**). There were significant changes in lung volume demonstrated with CIA+LPS exposure, but not LPS and CIA alone, as compared to Sham at 5 weeks (**Figure 1D**). In contrast, lung density (HU) increased significantly in both CIA and CIA+LPS mice vs. their respective baseline measurements (**Figures 1B, C**) as well as compared to Sham and LPS (**Figure 1D**).

### CIA+LPS co-exposure results in a decrease in lung compliance, increase in airway resistance and adverse lung pathology

**Figure 2:**
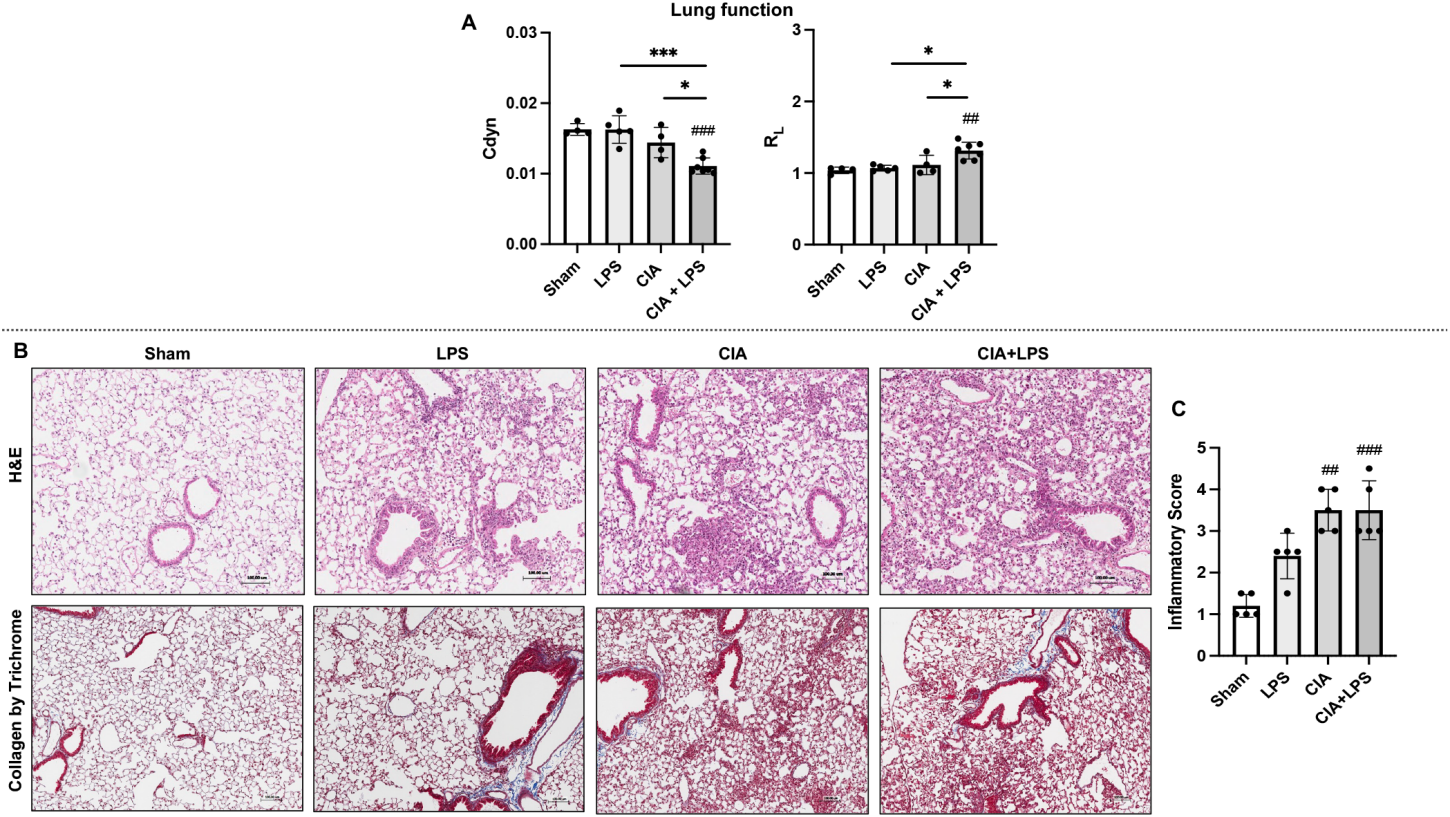
CIA+LPS co-exposure results in a decrease in lung compliance, increase in airway resistance and adverse lung pathology. **(A)** Lung function as determined by invasive pulmonary function testing represented by dynamic compliance (Cdyn) and total lung resistance (R_L_) at 5-week across treatment groups. **(B)** Representative H&E and trichrome stained lung images from one mouse per treatment group with corresponding scatter plot of **(C)** semiquantitative inflammatory scores. All data expressed as mean with SD bars of n=5 mice/group. *#* represent statistical significance versus Sham or baseline and brackets with * between groups. ^###,***^0.0001 to 0.001; ^##^0.001 to 0.1, ^*^0.01 to 0.05.

Following final micro-CT imaging, the same mice were invasively assessed for lung function using a small animal ventilator. Dynamic compliance (Cdyn) was significantly reduced in CIA+LPS mice compared to all other treatment groups (**Figure 2A**). Correspondingly, total airway resistance (R_L_) was significantly increased in CIA+LPS mice compared to all other experimental groups (**Figure 2A**).

Lung sections were next evaluated for histopathological changes by H&E and collagen deposition was evaluated by Masson’s trichrome staining (**Figure 2B**). There were increases in semi-quantitative inflammatory scores across treatment groups reaching statistical significance for CIA (p=0.0009) and CIA+LPS (p=0.0012) but not LPS (p=0.16) vs. Sham (**Figure 2C**). Arthritis inflammatory scores (mean ± SD) at 5 weeks were increased for LPS (0.4 ± 0.2, p=0.019), CIA (0.75 ± 0.5, p=0.0001), and CIA+LPS (1.1 ± 0.5, p<0.0001) vs. Sham (0 ± 0).

Collectively, these studies laid the foundation for subsequent studies aimed at delineating critical cells with corresponding gene expression data through high-throughput analyses.

### Single-cell RNA-seq identifies 16 unique immune cell subsets with CIA and LPS driving unique distributions of immune cells within clusters

**Figure 3:**
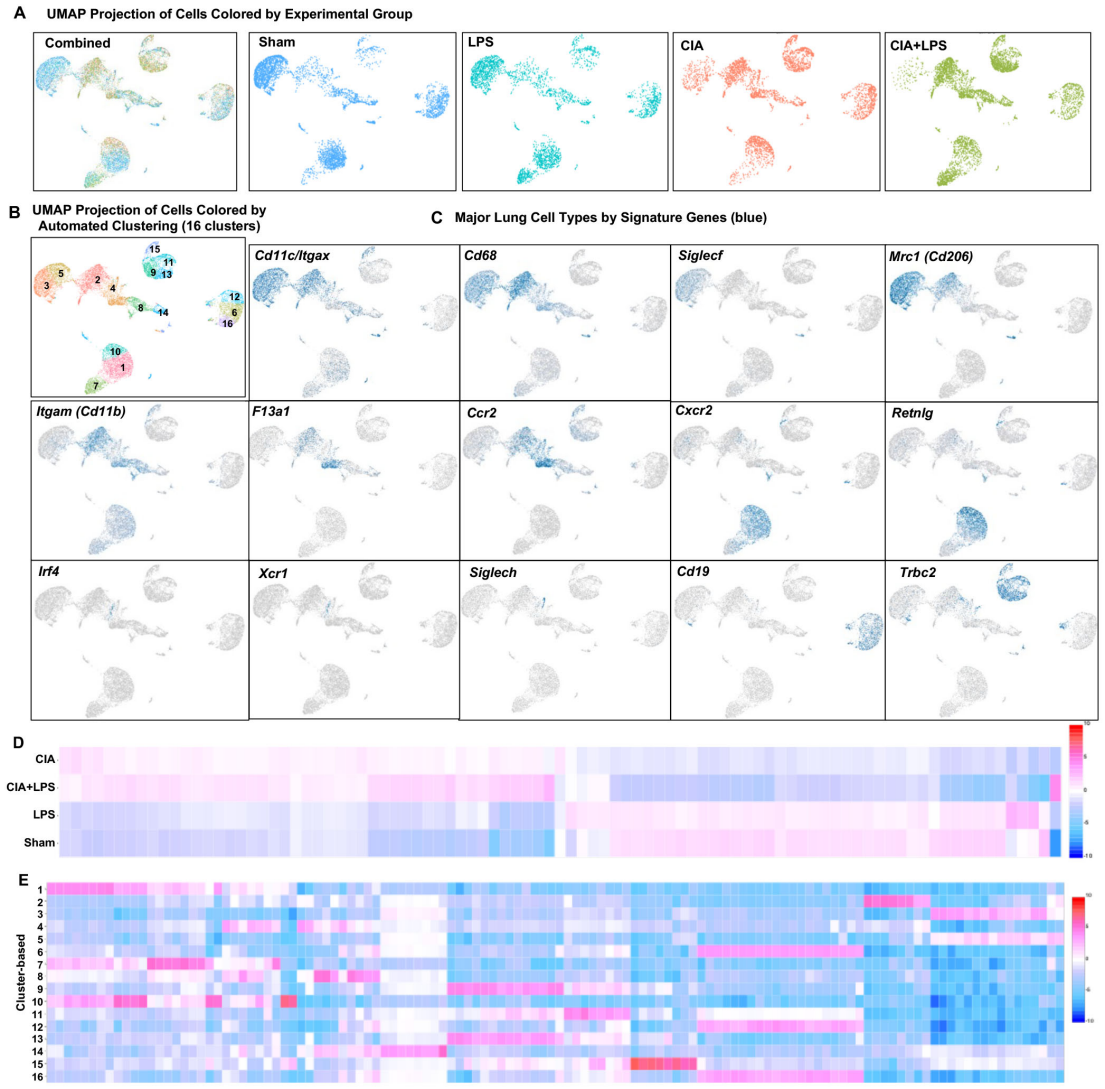
Unsupervised single-cell transcriptional profiling of lung CD45^+^ cells demonstrate differences in distribution of aggregated gene clusters with identification of 16 unique clusters among experimental groups. **(A)** UMAP plot with all the treatment groups by color merged (combined) and experimental groups plotted individually to show cell distribution. **(B)** UMAP plot demonstrating the 16 unique unsupervised clusters identified with **(C)** major lung cell types identified by signature genes including *Cd11c/Itgax, Cd68, Siglecf, Mrc1* (macrophages), *F13a1, Ccr2* (monocytes), *Cxcr2, Retnlg* (neutrophils), *Irf4, Xcr1*, *Siglech* (dendritic cells), *Cd19* (B cells), and *Trbc2* (T cells). **(D)** Heatmap demonstrates relationships by treatment-based groups based upon the top 20-25 genes per group. **(E)** Heatmap demonstrates relationships between the top 3 to 7 genes that distinguish each unique cluster from every other cluster. N=4-5 mice/group pooled per sample.

To cumulatively capture lung immune cells, the 10x genomics platform was utilized with a total of 16,748 cells analyzed (Sham: 4467, LPS: 3220, CIA: 3930, and CIA+LPS: 5131 cells). Unsupervised clustering of UMAP-projected CD45^+^ cells revealed 16 unique immune cell subsets differentially distributed by treatment group (**Figure 3A**) and graphically shown using unique colors and arbitrary numbers (**Figure 3B**). Major lung cell types were identified by signature genes (**Figure 3C**). Common gene markers of murine lung macrophages include *Itgax* (*Cd11c*), *Cd68*, *Siglecf*, and *Cd206* (45–47, 56). *Cd11b* gene is predominately expressed by myeloid-derived cells including macrophages, monocytes, and neutrophils (57). Thus, macrophage cells were identified for clusters 2, 3, and 5 and partially 4 and 8, noting gene expression of *Itgax/Cd11c* (clusters 2, 3, 5, partially 4 and 8), *Cd68* (clusters 2, 3, 5, partially 4 and 8), *Siglecf* (clusters 3,5), and *Cd206* (clusters 2, 3, 5, partially 4). *Cd11b* was expressed in clusters 2, 4, 8 as well as 1, 7, and 10 (the neutrophil cluster). The *F13a1* gene, which encodes for Factor XIIIA, has been linked to inflammatory-like monocytes with strong association with monocytes differentiating into interstitial macrophages as well as monocytes promoting and facilitating monocyte migration, adhesion, and phagocytosis within inflamed tissues (58, 59). CCR2 is a critical facilitator of monocyte recruitment and activation that upon reaching inflamed tissue can differentiate into macrophages (52, 53). Thus, inflammatory-like monocytes were identified with *F13a1* and *Ccr2* expression in clusters 2 and 4. (Note, *Ccr2* expression was also demonstrated in lymphocyte clusters 9, 13, 15, recognizing that lymphocytes can also express *Ccr2* (60, 61)). Clusters 1, 7 and 10 were identified as neutrophil subsets based on distribution of *Cxcr2* and *Retnlg* expression (62). Dendritic cells (DCs) were sparse, with conventional DCs characterized by *Irf4* and *Xcr1* gene expression (45, 63) and plasmacytoid DCs characterized by *Siglech* expression (64) located within cluster 4. Clusters 9, 11, 13, and 15 were identified as T lymphocytes based on *Trbc2* expression, and clusters 6, 12, and 16 were identified as B lymphocytes based on *Cd19* expression. The UMAP-projected pattern of immune cell infiltrates of CIA+LPS co-exposure was similar to that of CIA alone as opposed to LPS alone or Sham. In addition, a heatmap was generated by experimental group to show that the top 20-25 genes for CIA and CIA+LPS showed similar patterns that differed from LPS and Sham (**Figure 3D**). Next, the pattern of expression of the top 3 to 7 genes that distinguished each unique cluster from every other cluster are shown (**Figure 3E**). All significant gene expressing data is summarized for unique sample-based gene features (**Supplementary Data Files 1–3**), treatment group gene features as compared to Sham (**Supplementary Data File 2**), and unique cluster-based gene features (**Supplementary Data File 3**).

Notably, immune cell cluster 2, which expresses *Itgax* (*CD11c), Cd68, Mrc1 (Cd206), Itgam (Cd11b)*, *F13a1*, and *Ccr2* was strikingly increased with both CIA+LPS and CIA (vs. Sham and LPS), suggesting an importance for this infiltrating inflammatory and/or activated macrophage-monocyte subpopulation. Top/unique genes expressed in cluster 2 included *C1qc, C1qb, C1qa, Saa3, MS4a7, Apoe, Cxcl16*, and *Cadm1*. Cluster 4 (monocytic-like with high *Cd11b, F13a1* and *Ccr2*) was also increased, but to a lesser degree, with CIA+LPS and CIA. Top/unique genes expressed in cluster 4 included *Plac8, Plbd1, Ifitm3, Ifitm6, Ly6c2, F1da1, Ccr2, Ms4a4c*. Cluster 7, attributed to the neutrophil cluster expressing the *Cxcr2* gene, was predominant in both CIA+LPS and LPS (vs. Sham and CIA). Top/unique genes expressed in cluster 7 included *Rsad2, Ccl3, Ccl4, Pde1a, Ifit3b, Ifit1, Ifit3, Ltf, and Cxcl10*. Finally, T lymphocytes were increased with both CIA and CIA+LPS with predominant increases particularly observed for clusters 9 and 13 of CIA+LPS that included the following high expressing unique genes: *Cxcr6, IL17a, Ifng, Il1r1, Nebl, ctla4, Trdc, Cd5, Lat, IL19r1, Cd3g, Icos*, and *Sf6galnac3.*

Thus, in the following set of studies, we focused on delineating the immunophenotype of the lung monocyte-macrophage subpopulations by cell surface protein expression (as opposed to gene expression) to further characterize their role in CIA+LPS co-exposure by flow cytometry analysis.

### Co-exposure with CIA+LPS increases total cells and neutrophils and modulates CD11c^+^CD206^+^CD11b^var^ alveolar macrophages toward an activated status.

**Figure 4:**
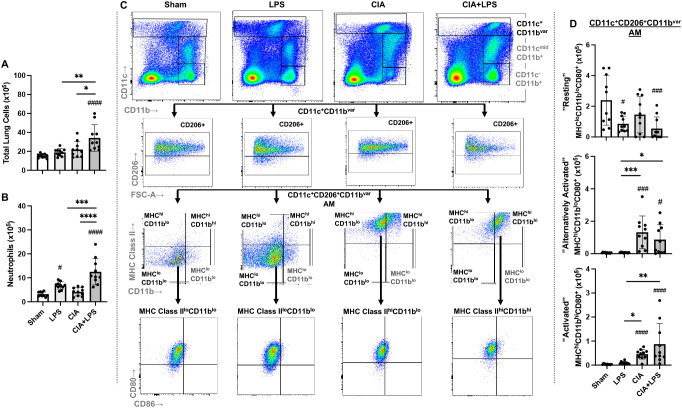
Lung total cell, neutrophil and CD11c^+^CD206^+^CD11b^var^ macrophage subpopulations vary by specific treatment group. **(A)** Total lung cells and **(B)** Ly6G^+^ neutrophils enumerated. **(C)** Representative contour plot highlighting the CD11c^+^CD11b^var^ macrophage subpopulations by one mouse per treatment group after removal of neutrophils gated from live CD45^+^ cells after excluding debris and doublets that are further delineated by CD206, MHC Class II and CD11b expression and costimulatory CD80 and CD86 expression. **(D)** CD11c^+^CD206^+^CD11b^var^ macrophage subpopulations defined as resting (MHC Class II^lo^, CD11b^lo^, CD80^+^), alternatively activated (MHC Class II^hi^, CD11b^lo^, CD80^+^), and activated (MHC Class II^hi^, CD11b^hi^, CD80^+^), differ across treatment groups. Scatter dot plots depict mean with SD bars of n=10 mice/group. *#* represent statistical significance versus Sham and brackets with * between groups. ^####,****^<0.0001; ^###,***^0.0001 to 0.001; ^**^0.001 to 0.1, ^#,*^0.01 to 0.05.

CIA+LPS co-exposure significantly increased total lung cell infiltrates vs. Sham, LPS, and CIA (**Figure 4A**). Neutrophil infiltrates (defined as CD11c^-^Ly6G^+^) were also significantly increased in CIA+LPS vs. Sham, LPS, and CIA with LPS exposure in isolation also increasing neutrophil infiltrates vs. Sham (**Figure 4B**). As outlined in the *Methods* section, monocyte-macrophage cell subpopulations were first broadly delineated based upon CD11c and CD11b expression into 3 groups: CD11c^+^CD11b^var^, CD11c^mid^CD11b^+^, and CD11c^-^CD11b^+^ cells. Alveolar macrophages (AMs) were defined as CD11c^+^CD206^+^CD11b^var^ (45–47) (**Figure 4C**). Moreover, these cells lacked expression of Ly6C and had high expression of the monocyte and macrophage marker F4/80 (ADGRE1) (65) (**Supplementary Figure 3**) as well as high expression of CD80 (**Figure 2C**) as AMs constitutively express CD80 (66). Here, we define 3 AM subpopulations: resting AMs (MHC Class II^lo^CD11b^lo^CD80^+^), alternatively activated AMs (MHC Class II^hi^CD11b^lo^CD80^+^), and activated AMs (MHC Class II^hi^CD11b^hi^CD80^+^) (**Figure 4C**).

Overall, there was a decrease in resting AMs with LPS, CIA, and CIA+LPS treatment as compared to Sham. Instead, there was an increase in alternatively activated AMs and activated AMs with CIA and CIA+LPS vs. Sham (and vs. LPS) (**Figure 4D**).

### Co-exposure CIA+LPS induces high MHC Class II expression across both high and low Ly6C subpopulations of CD11c^mid^CD206^-^CD11b^+^ interstitial macrophages

**Figure 5:**
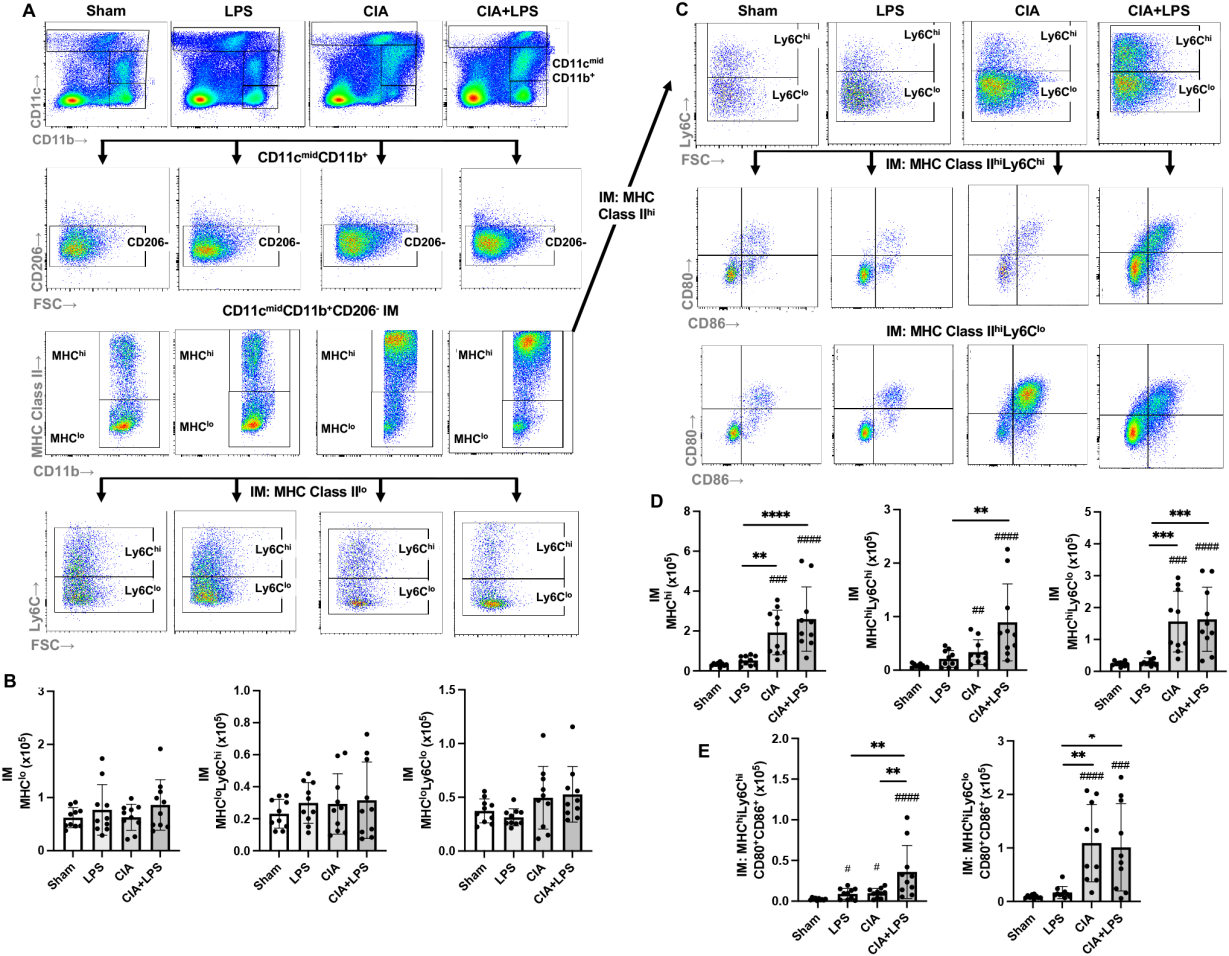
Lung CD11c^mid^CD11b^+^ interstitial macrophage subpopulations vary by specific treatment group. **(A)** Representative contour plot from one mouse per treatment group highlighting the CD11c^mid^CD11b^+^ interstitial macrophages (IM) after removal of neutrophils gated from live CD45^+^ cells debris and doublets that are further delineated by CD206 and MHC Class II with Ly6C expression of MHC Class II^lo^-expressing cells. **(B)** There is no difference in number of CD11c^mid^CD11b^+^CD206^-^MHC Class II^lo^ interstitial macrophages across treatment groups and correspondingly, there is no difference in number of these cells when further delineated by high or low Ly6C expression. **(C)** CD11c^mid^CD11b^+^MHC Class II high expressing cells were further delineated by Ly6C expression followed by CD80 and CD86 expression. **(D)** CD11c^mid^CD11b^+^CD206^-^MHC Class II^hi^ expressing interstitial macrophages and those cells with/without Ly6C expression cells were enumerated across treatment groups. **(E)** Double positive (CD80^+^CD86^+^) of MHC Class II^hi^ expressing macrophages were enumerated based on high/low Ly6C expression across treatment groups. (*Cell number distribution across treatment groups based upon CD80/CD86 expression is shown in* **Table 1**). Scatter dot plots depict mean with SD bars of n=10 mice/group. *#* represent statistical significance versus Sham and brackets with * between groups. ^####,****^<0.0001; ^###,***^0.0001 to 0.001; ^##,**^0.001 to 0.1, ^#,*^0.01 to 0.05.

The CD11c^mid^CD206^-^CD11b^+^ interstitial macrophage (IM) subpopulation was further delineated and enumerated by MHC Class II expression followed by Ly6C expression and CD80/CD86 expression across all treatment groups (**Figures 5A–E**). There was no difference across Sham, LPS, CIA, and CIA+LPS groups in MHC Class II low expressing IMs, regardless of Ly6C high vs. low expression status (**Figures 5A, B**). However, there were significant and robust increases in MHC Class II high-expressing IMs with CIA (5.8-fold increase) and CIA+LPS (7.9-fold increase) vs. Sham (**Figure 5C**). MHC Class II^hi^ IMs were then further characterized by Ly6C expression (**Figure 5C**). As compared to Sham, there were increases in both MHC Class II^hi^Ly6C^hi^ IMs and MHC Class II^hi^Ly6C^lo^ IMs (**Figure 5D**) with CIA (4.3-fold and 6.4-fold increase, respectively) and CIA+LPS (11.5-fold and 6.7-fold increase, respectively). These IM subpopulations were further delineated by co-stimulatory CD80 and CD86 expression (**Figure 5C**). There were significant increases in double CD80^+^/CD86^+^ expressing MHC Class II^hi^Ly6C^hi^ IMs for CIA+LPS vs. Sham (14.8-fold increase), vs. LPS (4.1-fold increase), and vs. CIA (3.6-fold increase) (**Figure 5E**). As compared to shame, there were also significant increases, but to a lesser degree, for these MHC Class II^hi^Ly6C^hi^ IMs for LPS (3.7-fold increase) and CIA (4.1-fold increase). For double CD80^+^/CD86^+^-expressing MHC Class II^hi^Ly6C^lo^ cells, there were increases with both CIA and CIA+LPS treatment groups vs. Sham and vs. LPS (**Figure 5E**). Complete expression patterns by co-stimulatory molecules (CD80, CD86, double positive and double negative) of the MHC Class II^hi^ IMs subpopulations by Ly6C expression are shown in **Table 1**.

**Table 1:** Expression patterns of co-stimulatory molecules of CD11c^mid^CD206^-^CD11b^+^ interstitial macrophage (IM) subpopulations.

	Sham	LPS	CIA	CIA+LPS
MHC^hi^Ly6C^hi^				
CD80^+^	0.033 (0.017)	0.102 (0.069)^#^	0.118 (0.068)^#^	0.401 (0.110)^#,*,†^
CD86^+^	0.035 (0.021)	0.139 (0.108)^#^	0.152 (0.110)^#^	0.539 (0.477)^#,†^
CD80^+^CD86^+^	0.0243 (0.128)	0.0893 (0.067)^#^	0.098 (0.058)^#^	0.358 (0.326)^#,*,†^
CD80^-^CD86^-^	0.034 (0.018)	0.094 (0.073)	0.166 (0.114)^#^	0.384 (0.358)^#,*^
MHC^hi^Ly6C^lo^				
CD80^+^	0.111 (0.039)	0.183 (0.114)	1.172 (0.769)^#,^^	1.075 (0.853)^#,†^
CD86^+^	0.114 (0.040)	0.207 (0.119)	1.274 (0.844)^#,^^	1.240 (0.932)^#,†^
CD80^+^CD86^+^	0.091 (0.033)	0.170 (0.113)	1.090 (0.722)^#,^^	1.012 (0.816)^#,†^
CD80^-^CD86^-^	0.110 (0.032)	0.0789 (0.022)	0.208 (0.088)^^^	0.330 (0.208)^#,*,†^

N=10 mice/group. Mean (SD).

Statistical significance (p<0.05) denoted as #vs. sham; *CIA+LPS vs. CIA, †CIA+LPS vs. LPS, ^CIA vs. LPS.

### Co-exposure CIA+LPS increased lung CD11c^-^CD206^-^CD11b^+^ monocytic-like cell subpopulations defined by high MHC Class II and/or high Ly6C expression

**Figure 6:**
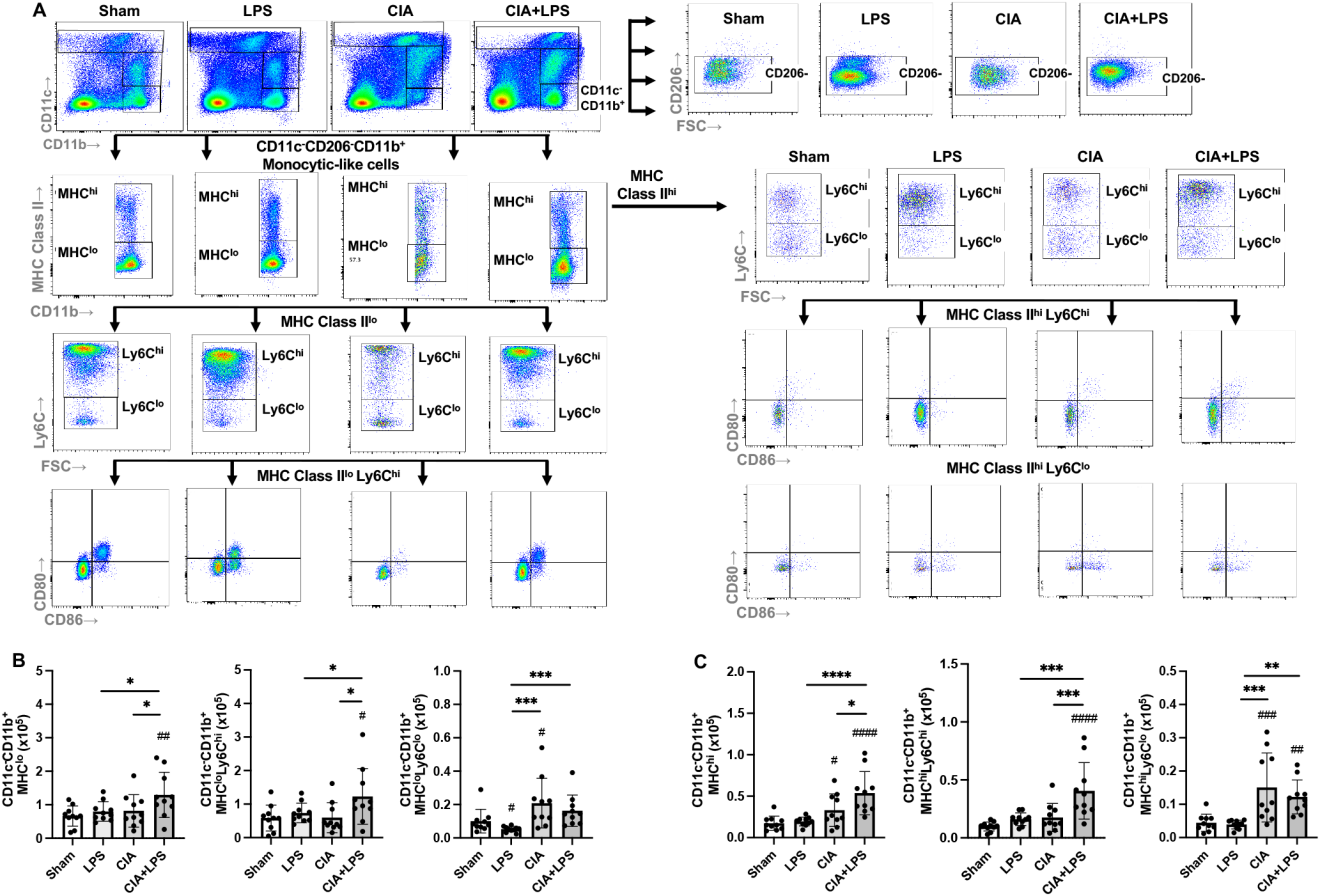
Lung CD11c^-^CD11b^+^ monocytic-like cell subpopulations vary by specific treatment group. **(A)** Representative contour plot from one mouse per treatment group highlighting the CD11c^-^CD11b^+^ monocytic-like cells after removal of neutrophils gated from live CD45^+^ cells debris and doublets that are further delineated in sequential manner by CD206, MHC Class II, Ly6C, and CD80/CD86 expression. Numbers of monocytic-like subpopulations based upon **(B)** MHC Class II^lo^ and Ly6C expression and **(C)** MHC Class II^hi^ and Ly6C expression enumerated across treatment groups. (*Cell number distribution across treatment groups based upon CD80/CD86 expression is shown in* **Table 2**). Scatter dot plots depict mean with SD bars of n=10 mice/group. *#* represent statistical significance versus Sham and brackets with * between groups. ^####,****^<0.0001; ^###,***^0.0001 to 0.001; ^##,**^0.001 to 0.1, ^#,*^0.01 to 0.05.

The CD11c^-^CD206-CD11b^+^ monocytic-like cell subpopulation was further delineated by MHC Class II expression followed by delineation by Ly6C and CD80/CD86 expression across treatment groups (**Figure 6A**). Overall, there was a lower frequency of MHC Class II^hi^ vs. MHC Class II^lo^ monocytic-like cells (**Figures 6B, C**). CIA+LPS co-exposure increased the number of MHC Class II^lo^Ly6C^hi^ monocytic-like cells as compared to all other treatment groups (**Figure 6B**). Whereas there was no difference between CIA+LPS vs. Sham for MHC Class II^lo^Ly6C^lo^ monocytic-like cells, this cell subpopulation was increased with CIA alone.

For the MHC Class II^hi^ monocytic-like cells, these cells were increased for both CIA and CIA+LPS (vs. Sham), but there were only increases in MHC ClassII^hi^Ly6C^hi^ for CIA+LPS as compared to all other groups (e.g., a 4-fold increase vs. Sharm) (**Figure 6C**). The MHC ClassII^hi^Ly6C^lo^ cells were increased for both CIA and CIA+LPS. The expression patterns by co-stimulatory molecules (CD80, CD86, double positive and double negative) of these monocytic-like subpopulations are summarized in **Table 2**.

**Table 2:** Expression patterns of co-stimulatory molecules of CD11c^-^CD206^-^CD11b^+^ monocytic-like cell subpopulations.

	Sham	LPS	CIA	CIA+LPS
MHC^hi^Ly6C^hi^				
CD80^+^	0.008 (0.005)	0.034 (0.036)^#^	0.026 (0.022)^#^	0.046 (0.033)^#^
CD86^+^	0.013 (0.008)	0.055 (0.058)^#^	0.034 (0.024)^#^	0.079 (0.059)^#^
CD80^+^CD86^+^	0.005 (0.003)	0.029 (0.037)	0.019 (0.018)	0.034 (0.028)^#^
CD80^-^CD86^-^	0.085 (0.036)	0.113 (0.050)	0.122 (0.072)	0.316 (0.228)^#,*,†^
MHC^hi^Ly6C^lo^				
CD80^+^	0.005 (0.002)	0.009 (0.005)^#^	0.009 (0.006)	0.015 (0.009)^#^
CD86^+^	0.010 (0.005)	0.015 (0.008)	0.054 (0.047)^#,^^	0.0538 (0.036)^#,†^
CD80^+^CD86^+^	0.002 (0.001)	0.006 (0.004)^#^	0.006 (0.005)^#^	0.011 (0.008)^#^
CD80^-^CD86^-^	0.032 (0.026)	0.021 (0.009)	0.093 (0.077)^#,^^	0.064 (0.025)^#,†^
MHC^lo^Ly6C^hi^				
CD80^+^	0.214 (0.197)	0.186 (0.066)	0.078 (0.080)^#,^^	0.199 (0.160)
CD86^+^	0.225 (0.143)	0.214 (0.076)	0.148 (0.162)	0.301 (0.311)
CD80^+^CD86^+^	0.145 (0.103)	0.149 (0.056)	0.071 (0.078)	0.151 (0.111)
CD80^-^CD86^-^	0.289 (0.192)	0.485 (0.216)	0.442 (0.304)	0.878 (0.487)^#,*,†^
MHC^lo^Ly6C^lo^				
CD80^+^	0.035 (0.016)	0.026 (0.009)	0.034 (0.027)	0.019 (0.020)
CD86^+^	0.052 (0.022)	0.032 (0.014)	0.041 (0.032)	0.027 (0.030)^#^
CD80^+^CD86^+^	0.034 (0.016)	0.024 (0.009)	0.031 (0.025)	0.017 (0.019)^#^
CD80^-^CD86^-^	0.047 (0.065)	0.019 (0.006)	0.164 (0.150)^#,^^	0.135 (0.102)^#,†^

N=10 mice/group. Mean (SD).

#vs. sham; *CIA+LPS vs. CIA, †CIA+LPS vs. LPS, ^CIA vs. LPS.

### Unique clustering by tSNE algorithm of monocytes-macrophages by treatment group highlight robust MHC Class II and Ly6C expression with co-exposure CIA+LPS

**Figure 7:**
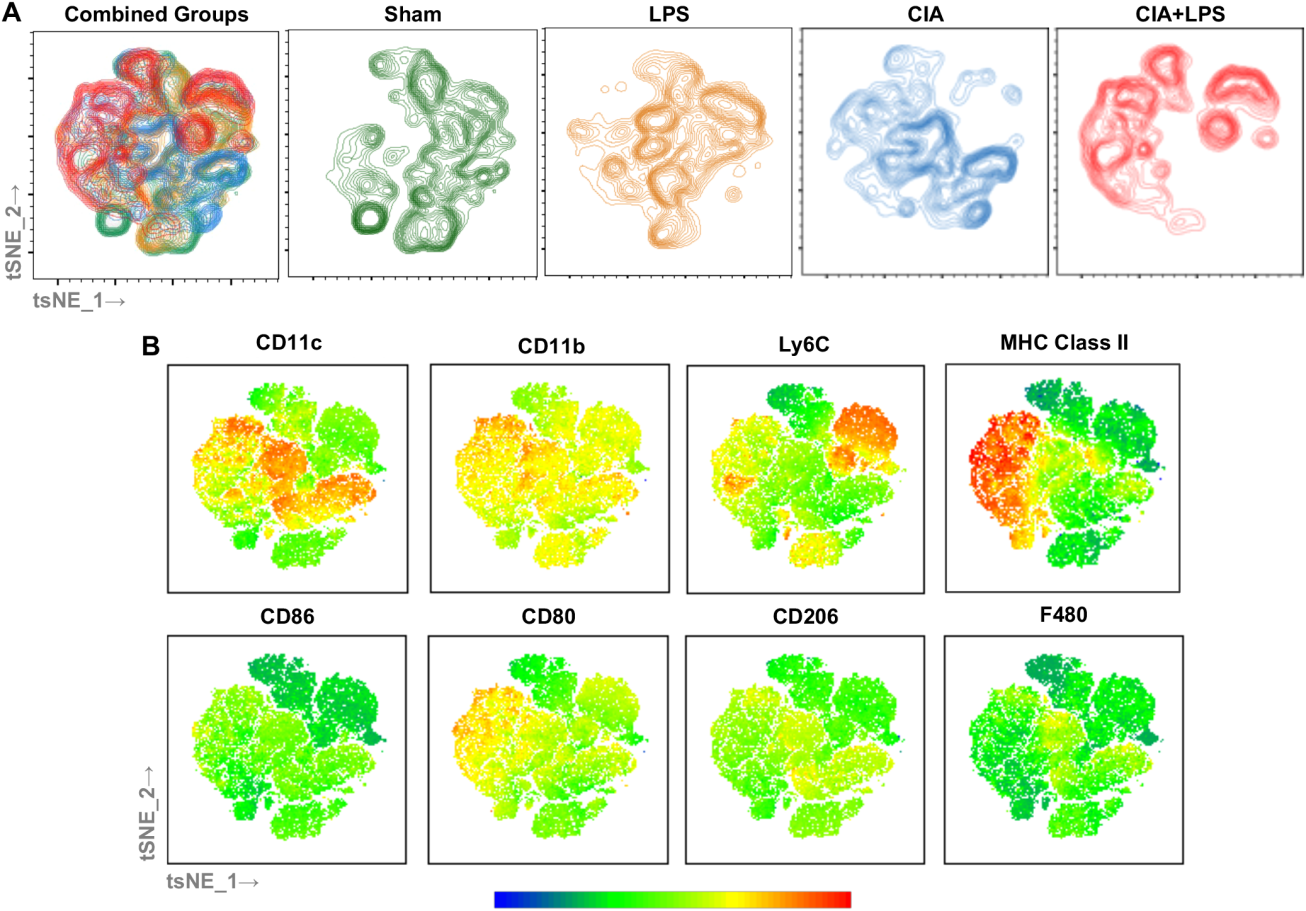
Clustering by tSNE algorithm of lung monocytes-macrophages from representative of each Sham, LPS, CIA, and CIA+LPS treatment group. **(A)** tSNE mapping of the data applied to non-debris, single, live CD45^+^Ly6G^-^lymphocyte gate^-^ cells (30,000 cells/sample) of a representative mouse lung combined and from each group (sham, LPA, CIA, CIA+LPS). **(B)** Multigraph overlays of staining intensity (blue: low and red: high) of cell surface markers utilized for delineating lung monocyte-macrophage subpopulations demonstrated by contour plots.

To provide visualization of the complex multi-dimensional data, the tSNE algorithm was applied to CD45^+^, neutrophil (Ly6G) negative, lymphocyte gate negative cells, combined and separated from a representative murine lung from each group (**Figure 7A**). The embedded tSNE mapping is demonstrated by contour plot with overlay colors by expression intensity, labeling cell marker expression from the combined map to provide visualization of marker expression differences (**Figure 7B**). CIA+LPS co-exposure demonstrates unique distribution with a striking cellular cluster shift towards the left that is delineated by high MHC Class II and CD11c expression as well as a predominant top and rightward cluster delineated by high Ly6C expression. Collectively, these flow cytometric studies highlight that co-exposure CIA+LPS uniquely and robustly upregulates MHC Class II and Ly6C expression across the heterogenous lung monocyte-macrophage cell spectrum, and this would imply a polarization state consistent of cells with activated, inflammatory, and pro-fibrotic features (27, 46, 49).

### Phagocytic activity of lung monocytes-macrophages differs across treatment condition and subpopulation

**Figure 8:**
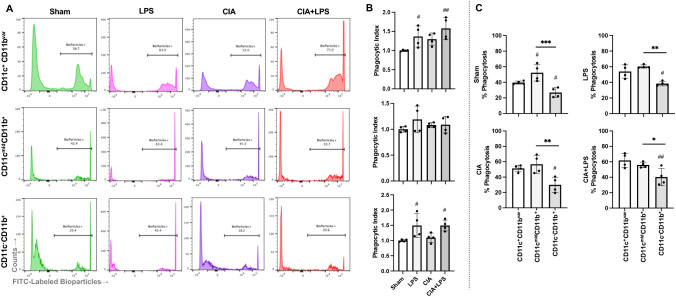
Phagocytic activity of lung monocyte-macrophage subpopulations across treatment groups. **(A)** Representative histograms from one mouse per treatment group depicting the phagocytic ability of the 3 generalized monocyte-macrophage subpopulations including CD11c^+^CD11b^var^, CD11c^mid^CD11b^+^, and CD11c^-^CD11b^+^ cells upon incubation with fluorescently labeled *E coli*. **(B)** Scatter plots with bars depict mean with SD of phagocytic index normalized to respective Sham cell population (% bioparticle positive per mouse divided by averaged Sham per experimental run). **(C)** % Phagocytosis (Bioparticle +) by treatment group across these 3 cell subpopulations. N=2-3 mice pooled per sample for 4 samples/group (N=10 mice/condition). ^***^0.0001 to 0.001; ^##,**^0.001 to 0.01, ^#,*^0.01 to 0.05.

Phagocytic ability reflects an important innate immune function for host clearance of lung particulates and debris that was assessed in the 3 broadly defined monocyte-macrophage subpopulations: AM (CD11c^+^CD11b^var^), IM (CD11c^mid^CD11b^+^), and monocytic-like cells (CD11c^-^CD11b^+^) (**Figures 8A, B**). Representative histograms are shown for all treatment groups for each cell subpopulation (**Figure 8A**) with the phagocytic index of 4 samples of pooled mice (n=10 total mice/condition) for all treatment groups across each of the 3 cell subpopulations (**Figure 8B**). There were significant increases in phagocytic ability of both AMs and monocytic-like cells with LPS and CIA+LPS but not CIA vs. Sham. There was no difference in phagocytic ability for IMs across treatment groups. Next, phagocytic ability differences within treatment groups across cell subpopulations were assessed (**Figure 8C**). Within Sham mice and as compared to AMs, IMs demonstrated increased phagocytic ability and monocytic-like cells demonstrated decreased phagocytic ability. As phagocytic ability was generally increased with treatment groups for AMs, there was no difference between the phagocytic ability of AMs to that of IMs for LPS, CIA, and CIA+LPS. For all experimental groups, the phagocytic ability of monocytic-like cells was reduced in comparison to AMs and/or IMs.

As these studies collectively highlight the potential role for monocyte-derived lung macrophages in CIA+LPS, the following set of studies sought to determine the functional consequence of reducing the infiltration of lung macrophages by depleting the reservoir of circulating monocytes focused on CIA+LPS co-exposed mice.

### Systemic clodronate liposome delivery reduces CIA+LPS co-exposure-induced airway and cellular influx

**Figure 9:**
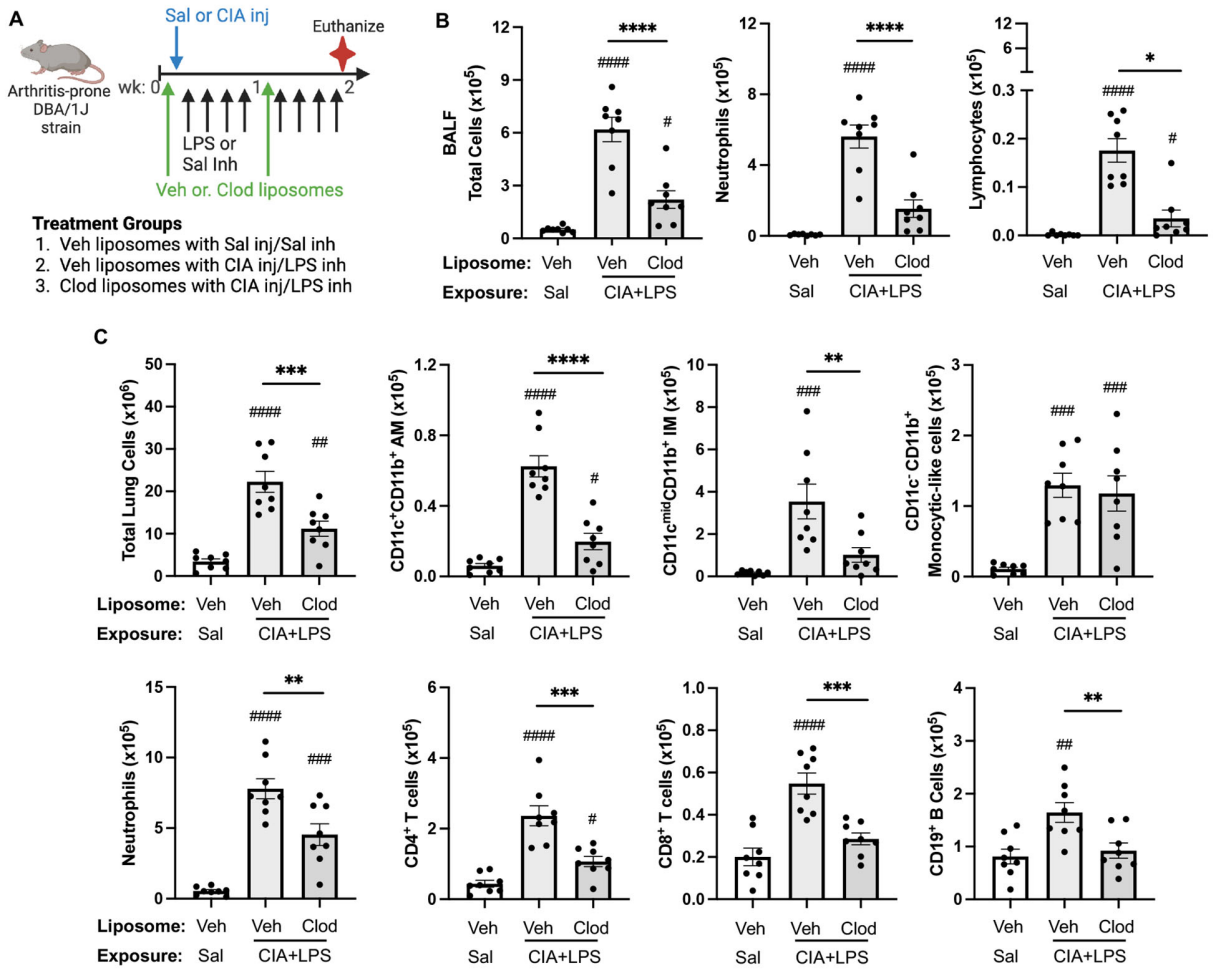
Systemic clodronate liposome delivery reduces 2-week CIA+LPS co-exposure-induced airway and lung tissue cellular influx. **(A)** Mice were treated with vehicle (Veh) control or clodronate (Clod) liposome twice (baseline and week 1), saline or CIA injection (inj) once at baseline, and then received intranasal inhalation with LPS (100 ng) or saline daily for 2 weeks (weekends excluded). **(B)** Bronchoalveolar lavage fluid (BALF) total cells, neutrophils, and lymphocytes and **(C)**, lung tissue cellular infiltrates. Total lung cells enumerated with number of lung cells determined by multiplying lung cell % gated population by flow cytometry by total lung cells enumerate from lung sample. AM, alveolar macrophages; IM, interstitial macrophages. All data expressed as mean with SD bars of n=8 mice/group. *Symbols* represent statistical significance versus vehicle liposome+saline exposures (#) or between CIA+LPS mice treated with vehicle versus clodronate liposomes (*). ^####,****^<0.0001; ^###,***^0.0001 to 0.001; ^##,**^0.001 to 0.1, ^#,*^0.01 to 0.05.

To reduce the circulating/systemic reservoir of available monocytes-macrophages, mice were treated with intravenous clodronate liposomes (vs. vehicle control liposomes) one day prior to initiation of CIA+LPS co-exposure modeling with a dose repeated 1-week later (**Figure 9A**). In these proof-of-concept studies, the exposure model was terminated at 2 weeks (as opposed to 5 weeks) because of unacceptable tail vein irritation and inflammation from repeated clodronate and CIA injections. In the CIA+LPS mice, there were significant reductions (% reduction) in airway influx (BALF) of total cells (64%), neutrophils (73%), and lymphocytes (80%) but not macrophages with clodronate liposomes vs. vehicle (**Figure 9B**). Likewise, there were significant reductions in lung tissue infiltrates of total lung cells (50%), activated AMs (CD11c^+^CD11b^+^, 68%), IMs (71%), neutrophils (42%), CD4^+^ T cells (55%), CD8^+^ T cells (48%), and CD19^+^ B cells (44%) but not monocytic-like cells in CIA+LPS mice treated with clodronate liposomes vs. vehicle liposomes (**Figure 9C**).

### CIA+LPS co-exposure induced mediators are reduced with systemic clodronate liposome delivery

**Figure 10:**
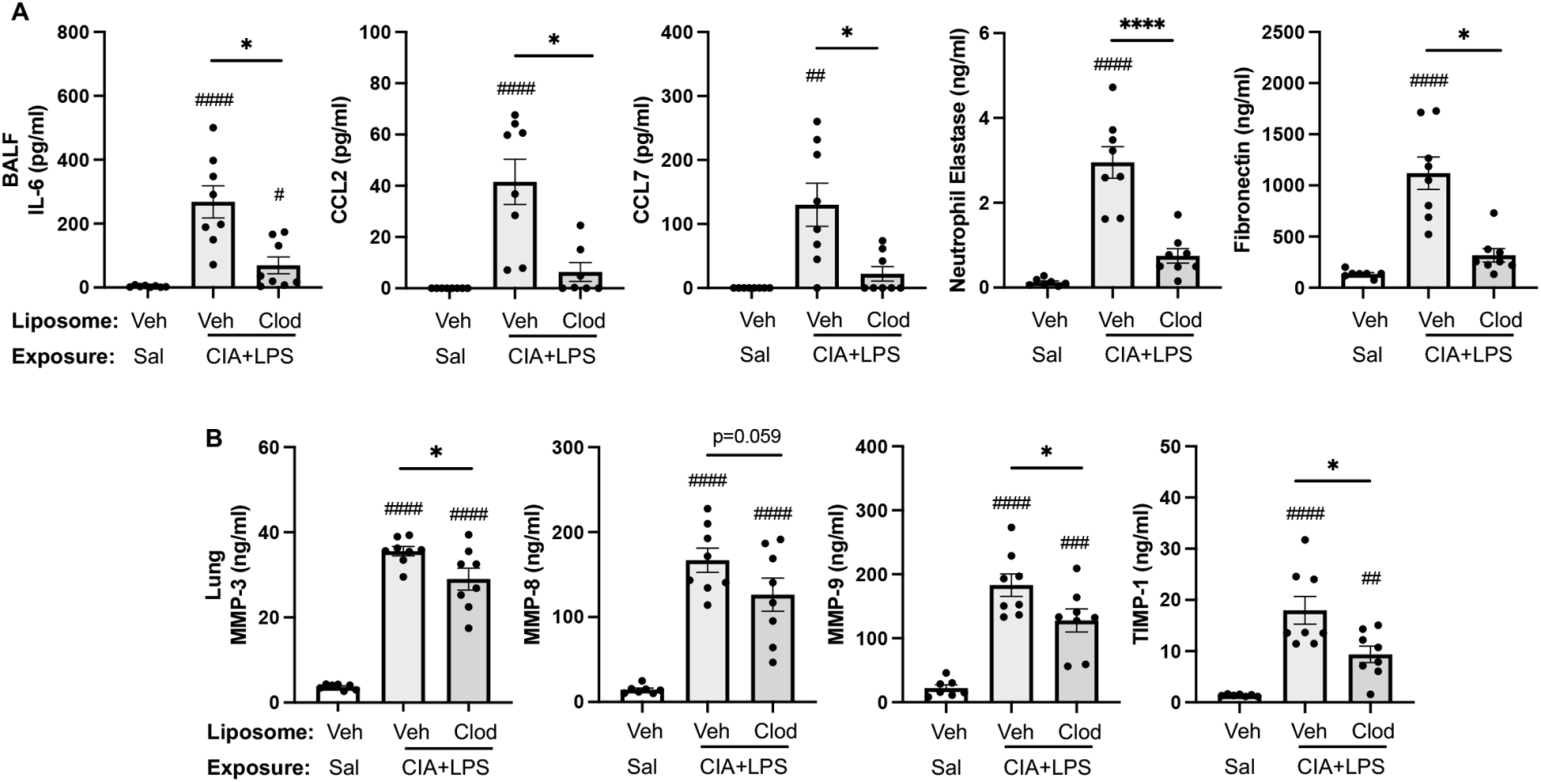
CIA+LPS co-exposure induced mediators reduced with systemic clodronate liposome delivery. **(A)** Bronchoalveolar lavage fluid (BALF) levels of IL-6, CCL2, CCL7, neutrophil elastase, and fibronectin. **(B)** Lung tissue homogenate levels of MMP-3, -8, -9, and TIMP-1. All data expressed as mean with SD bars of n=8 mice/group. *Symbols* represent statistical significance versus vehicle liposome+saline exposures (#) or between CIA+LPS mice treated with vehicle versus clodronate liposomes (*). ^####,****^<0.0001; ^###^0.0001 to 0.001; ^##^0.001 to 0.1, ^*^0.01 to 0.05.

Treatment with intravenous clodronate liposomes (vs. vehicle) also resulted in reductions (% reduction) in CIA+LPS-induced BALF levels of IL-6 (74%), CCL2 (85%, p=0.014), CCL7 (83%), neutrophil elastase (75%), and fibronectin (72%) (**Figure 10A**). There were also reductions in lung tissue homogenate levels of MMP-3 (18), MMP-9 (30%), and TIMP-1 (48%), in CIA+LPS mice treated with clodronate liposomes vs. vehicle liposomes (**Figure 10B**). However, there were no treatment differences between clodronate and vehicle in BALF levels of TNF-⍺ and CXCL2 or lung tissue homogenate levels of IL-6, TNF-⍺, CXCL2, CCL2, CCL7, neutrophil elastase, and fibronectin following CIA+LPS exposure (**Table 3**).

**Table 3:** CIA+LPS co-exposure induced mediators not significantly affected by systemic clodronate liposome delivery.

	Vehicle Liposome & Sal+Sal	Vehicle Liposome & CIA+LPS	Clodronate Liposome & CIA+LPS
BALF			
TNF-⍺, pg/ml	6.7 (5.0)	186 (91.9)^###^	74.5 (30.3)^#^
CXCL2, pg/ml	56.4 (27.0)	88.2 (48.4)	89.5 (48.4)
Lung tissue			
IL-6	27.6 (18.4)	85.7 (46.2)^##^	31.3 (41.1)
TNF-⍺, pg/ml	0.4 (0.7)	51.6 (24.9)^###^	28.7 (20.7)^##^
CXCL2, pg/ml	55.5 (25.1)	619.4 (254.8)^####^	278.9 (175.3)^#^
CCL2, pg/ml	98 (39)	1422 (797)^###^	1098 (814)^##^
CCL7, pg/ml	28 (37)	1795 (1206)^###^	877 (679)^##^
Neutrophil Elastase, ng/ml	2.3 (2.6)	21.1 (10.8)^###^	12.3 (5.2)^##^
Fibronectin, ng/ml	62.4 (18.4)	201.7 (49)^###^	161.3 (62)^##^

Mean (SD), n=8 mice/group except n=7 mice/vehicle liposome & saline exposures for lung fibronectin, neutrophil elastase, and CXCL2.

*#*represent statistical significance versus vehicle liposome & saline exposures. ^####^<0.0001; ^###^0.0001 to 0.001; ^##^0.001 to 0.1, ^#^0.01 to.005.

### Systemic clodronate liposome delivery reduces CIA+LPS co-exposure induced lung tissue inflammation, infiltrating CCR2^+^ monocyte-macrophages and MPO^+^ neutrophils as well as vimentin and lung CIT and MAA autoantigen expression

**Figure 11:**
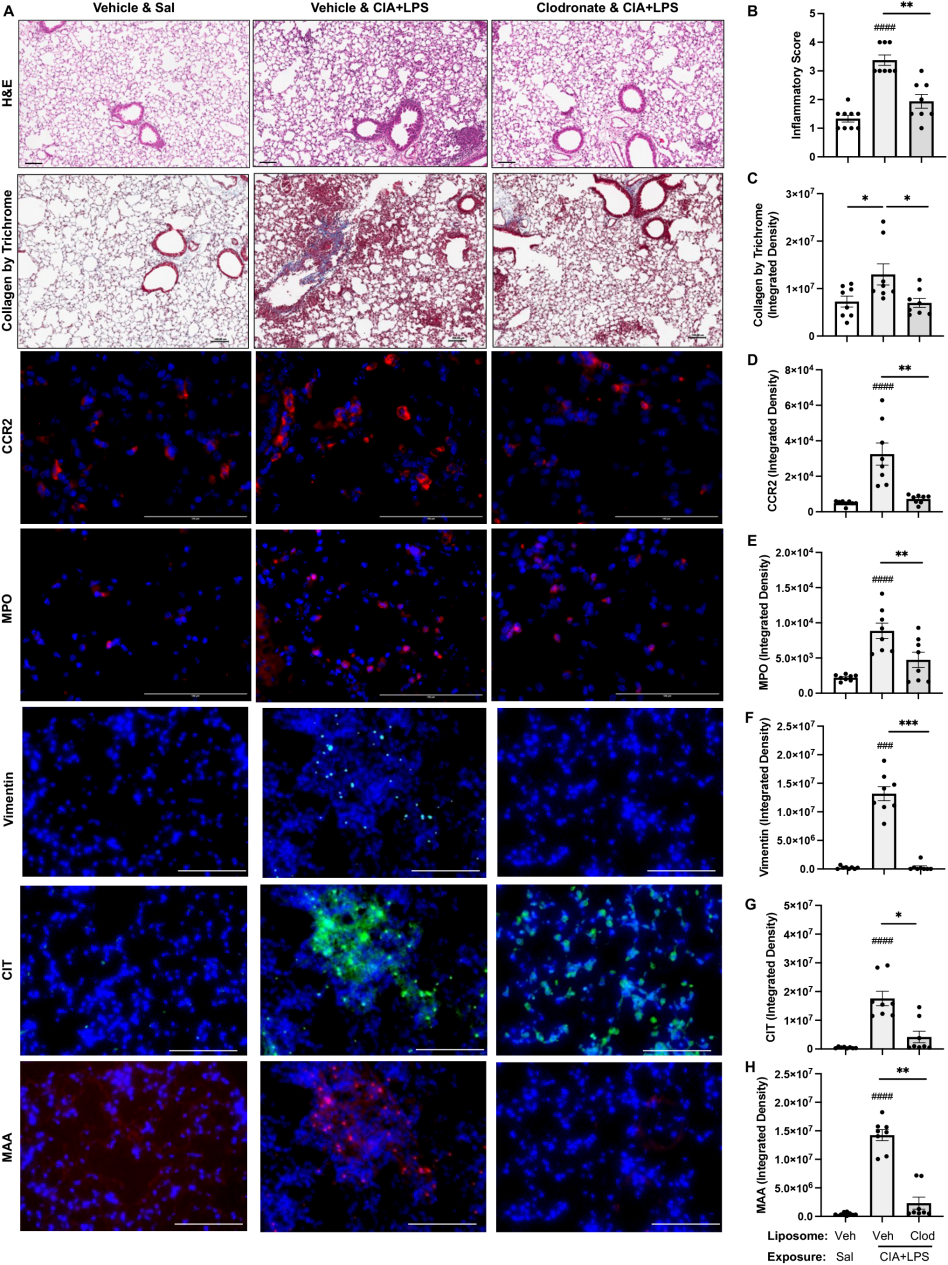
Systemic clodronate liposome delivery reduces CIA+LPS co-exposure induced lung tissue inflammation, collagen deposition, infiltrating CCR2^+^ monocyte-macrophage cells and myeloperoxidase (MPO)^+^ neutrophils, vimentin expression, and lung CIT and MAA autoantigen expression. **(A)** Representative images from treatment groups stained by H&E, collagen by trichrome, CCR2 (red), and MPO (red), vimentin (teal), citrulline (CIT, green), and malondialdehyde-acetaldehyde (MAA, red) modified proteins with DAPI nuclei staining (blue) by confocal microscopy. Scatter plots with bars depict mean with SD of **(B)** semi-quantitative lung inflammatory score and integrated density of **(C)** Collagen by trichrome, **(D)** CCR2 and **(E)** MPO, **(F)** vimentin, **(G)** CIT, and **(H)** MAA quantified per each mouse. Line scale denotes 100 μm. n=8 mice/group. *Symbols* represent statistical significance versus vehicle liposome+saline exposures (#) or between CIA+LPS mice treated with vehicle versus clodronate liposomes (*). ^####^<0.0001; ^###,***^0.0001 to 0.001; ^**^0.001 to 0.1, ^*^0.01 to 0.05.

Lung sections from mice treated with vehicle and clodronate liposomes with CIA+LPS co-exposure and control mice treated with vehicle liposomes and saline+saline were evaluated for histopathological changes by H&E, collagen by trichrome, CCR2^+^, and MPO^+^ cell infiltrates, vimentin expression, and expression of CIT and MAA autoantigens (**Figure 11A**). Semi-quantitative inflammatory scores following CIA+LPS exposure were significantly reduced with clodronate vs. vehicle control liposomes (**Figure 11B**). Correspondingly, CIA+LPS exposure-induced collagen deposition was reduced with clodronate vs. vehicle control liposome (**Figure 11C**). CIA+LPS-induced CCR2^+^ monocyte/macrophage cell infiltrates, quantified by flow cytometry, were reduced by 78% (**Figure 11D**) and MPO^+^ neutrophils were reduced by 53% (**Figure 11E**). Next, the expression of vimentin, an extracellular matrix protein that is also targeted by post-translational modifications generated during the process of inflammation and oxidative stress and expressed by monocyte/macrophages and lung mesenchymal cells, was reduced 98% in CIA+LPS mice treated with clodronate vs. vehicle (**Figure 11F**). Correspondingly, there were significant treatment-related reductions in the expression of lung autoantigens including reductions in the expression of CIT (76% reduction) and MAA (84% reduction) (**Figures 11G, H**).

## Discussion

RA-ILD represents the most overrepresented cause of death in RA (4, 67, 68). Knowledge of the critical cellular players, biomarkers of disease, and efficacious treatment options are limited, and the factors driving the development and progression of lung disease in RA are unclear. Studies herein demonstrate that the interaction of inhalation-induced airway inflammation and autoimmune arthritis resulted in lung disease associated with robust and activated monocyte/macrophages, which directly mediate the adverse lung consequences observed in this informative disease model. Moreover, CIA+LPS induced several subpopulations of activated AMs, recruited, inflammatory, and activated IMs, and activated/inflammatory monocytic-like cells, all sharing high expression levels of MHC Class II antigen, co-stimulatory molecule(s), and inflammatory markers (Ly6C, CD11b). Strikingly, IMs generated with CIA+LPS was more aligned to autoimmune arthritis induction than inhalant LPS exposure. Finally, the targeted depletion of circulating monocytes was associated with marked reductions in CIA+LPS-induced IMs, neutrophils, lymphocytes, inflammatory/pro-fibrotic mediators, and expression of vimentin and citrullinated and malondialdehyde acetaldehyde (MAA)-modified proteins/lung autoantigens. Collectively, the results of this study highlight the central role that infiltrating/recruited IMs appear to play in the pathogenesis of RA-ILD.

The complexity of the pathogenesis of RA-associated lung disease involves the dynamic interplay between environmental triggers and genetic susceptibility ultimately leading to a specific loss of tolerance and autoimmunity with increasing evidence suggesting that initial immune responses may be initiated at the level of pulmonary mucosa (69), noting that subclinical lung disease occurs in upwards of 30-40% of RA patients. In addition to measures of inflammation and histopathologic assessments, additional metrics were deployed in this study to better characterize lung disease in the CIA+LPS co-exposure animal model. Live micro-CT imaging revealed a gradual increase in non-aerated lung volume, a feature of lung fibrosis in mice, with corresponding increases in lung tissue density that were most apparent after 5 weeks. Interestingly, arthritis induction alone also increased lung density but not non-aerated lung volume, consistent with our data demonstrating increases in lung inflammation observed with CIA alone. Moreover, declines in lung function including reduced tissue compliance and increased resistance, were only observed in the co-exposed animals. Collectively, these imaging and functional studies support the use of this model as they align with observations demonstrated with other experimental lung fibrosis models including those employing bleomycin and silica.

In patients with RA-ILD, circulating monocytes demonstrate high expression of genes involved in macrophage differentiation-activation and pro-fibrosis processes (18). In this current study, single-cell RNA-seq studies identified a striking immune cell cluster (i.e., cluster 2) that was unique to CIA and CIA+LPS as opposed to Sham or LPS with a predominance of features characteristic of activated/inflammatory monocyte-derived macrophages. This observation coupled with human data of altered circulating monocytes in patients with RA-ILD may suggest that future approaches targeting and/or reducing lung monocyte/macrophage burden may be advantageous in RA lung disease. Moreover, complement genes were highly expressed with co-exposure CIA+LPS. Although dysregulation of the complement system appears to be an important factor in autoimmune and pulmonary disease and current therapies such as glucocorticoids, pirfenidone, and disease modifying antirheumatic drugs and targeted biologics may act indirectly on the complement system (70, 71), there are currently no therapies approved or recommended in RA-ILD that target the complement system. Other prominent monocyte/macrophage cluster specific genes expressed included interferon-related genes and genes involved with inflammation (*Apoe, Saa3*), cell adhesion (*Cadm1*), lipid metabolism (*Apoe, Pldbd1*), as well as those with less known immune cell functions involved in differentiation and activation (*Plac8, Ms4a4c, Ms4s7*) (72–75). Future studies may be warranted to further explore targeting these molecules and/or related signaling pathways for the development of novel approaches.

Macrophages in RA-ILD are heterogeneous and not only initiate the inflammatory response but also contribute to repair and resolution as these cells possess high plasticity and various immune functions (69). Without the environmental stimulant (i.e., inhalant endotoxin), our animal modeling studies demonstrated that systemic arthritis induction alone (i.e., CIA) strikingly increases MHC Class II expression across all monocyte-macrophage subpopulations in the lung with less robust expression of “inflammatory” and/or pro-fibrosis markers such as Ly6C (27, 49). High MHC Class II expression implies an increased potential for antigen presentation to T cells to potentially facilitate the activation of autoreactive T cells that target self-antigens (76). Thus, this suggests that CIA (and perhaps RA) alone acts to prime the lung for an adverse inflammatory response in the wake of an environmental stimulus with this “priming” effect mediated by monocytes. The addition of inhalant endotoxin potentiated monocyte/macrophage numbers, inflammatory marker expression, and phagocytic ability, underscoring that clinical lung disease development in RA likely requires multiple factors including environmental triggers. On the converse, it is well-recognized that repetitive endotoxin exposures are associated with chronic adaptation or tolerance responses; yet the current studies may imply that arthritis induction/autoimmunity may break endotoxin tolerance. Thus, combined modeling (CIA+LPS) strikingly potentiated/activated lung macrophages as compared to one-hit alone.

Depleting the reservoir of circulating monocytes in the setting of co-exposure suggests that systemic monocytes are a critical to orchestrating disease severity and progression. Monocyte-macrophage secretory products including IL-6 that contributes to the proliferation of T cells and inflammation/fibrosis (69) as well as leukocyte chemoattractants (CCL2 and CCL7), fibronectin and vimentin, were all markedly reduced in CIA+LPS mice following depletion of the circulating monocyte pool. Furthermore, there were robust reductions in both CIT- and MAA-modified protein generation following co-exposure with CIA+LPS resulting from the depletion of circulating monocytes. This is highly relevant as CIT and MAA stimulate undifferentiated macrophages and soluble factor secretion that drive an aggressive fibroblast phenotype and extracellular matrix deposition that characterize lung fibrosis (77–79). Moreover, these modifications have been implicated in promoting tolerance loss leading to disease-related autoimmunity (54, 69, 80). Although reductions were observed herein with the expression of extracellular matrix proteins, these reductions were relatively modest, suggesting that circulating and recruited monocytes/macrophages are not the major cellular source of these wound repair factors.

There are limitations to this study. This study focused on LPS as the inhalant exposure as it is commercially available, standardized to promote reproducibility, and is frequently contained in environmental/occupational exposures that have been implicated in RA (8–14, 81–83). Other airborne hazards relevant to RA-associated lung disease such as cigarette smoke, silica, complex air pollution/particulates, and chemical toxins/gases as well as combination of these exposures will need to be explored with and without systemic arthritis induction in future work. We also did not delineate and evaluate DCs; DCs are critical antigen presenting cells that also express high MHC Class II and co-stimulatory molecules as well as can express CD11c, CD11b, and Ly6C in various states, but uniquely express CD103 (as well Irf4, Xcr1, Siglec-H) and do not express CD206, CD68, and Siglec-F (45, 63, 64, 84–86), and as such, future studies are needed to careful delineate the smaller subset of DCs. Other potential future studies could include isolating specific cell populations to investigate *ex vivo* cellular and functional responses and/or the adoptive transfer of cells from a diseased mouse to a naïve mouse to determine whether adverse biological responses could be reproduced. Furthermore, direct depletion of lung macrophages by lung-delivery of clodronate liposomes at various time points in this co-exposure model, as opposed to systemic depletion, could also be investigated to determine functional consequences.

In conclusion, the interaction of inhalation-induced airway inflammation and autoimmune arthritis results in lung disease associated with uniquely activated infiltrating inflammatory monocytes-macrophages that mediate adverse lung consequences in mice. Whereas the induced IM immunophenotype is more aligned to arthritis induction than endotoxin exposure, co-exposure modeling offers unique features that informs RA-ILD pathogenesis that could be leveraged in future pre-clinical work. It will also be important for future studies to translate these observations to humans to fully understand the mechanisms and impact of the unique lung macrophage subpopulations of RA-ILD.

## Data Availability

The datasets presented in this study can be found in online repositories. The names of the repository/repositories and accession number(s) can be found in the article/**Supplementary Material**.
