# Supplementary figures and images for: Lung disease in relation to unique monocyte-macrophage subpopulations induced by combined inhalant endotoxin and collagen-induced arthritis
Source: Front Immunol. 2025 Apr 9;16:1557583. doi: 10.3389/fimmu.2025.1557583 (PMC12014730; doi:10.3389/fimmu.2025.1557583)

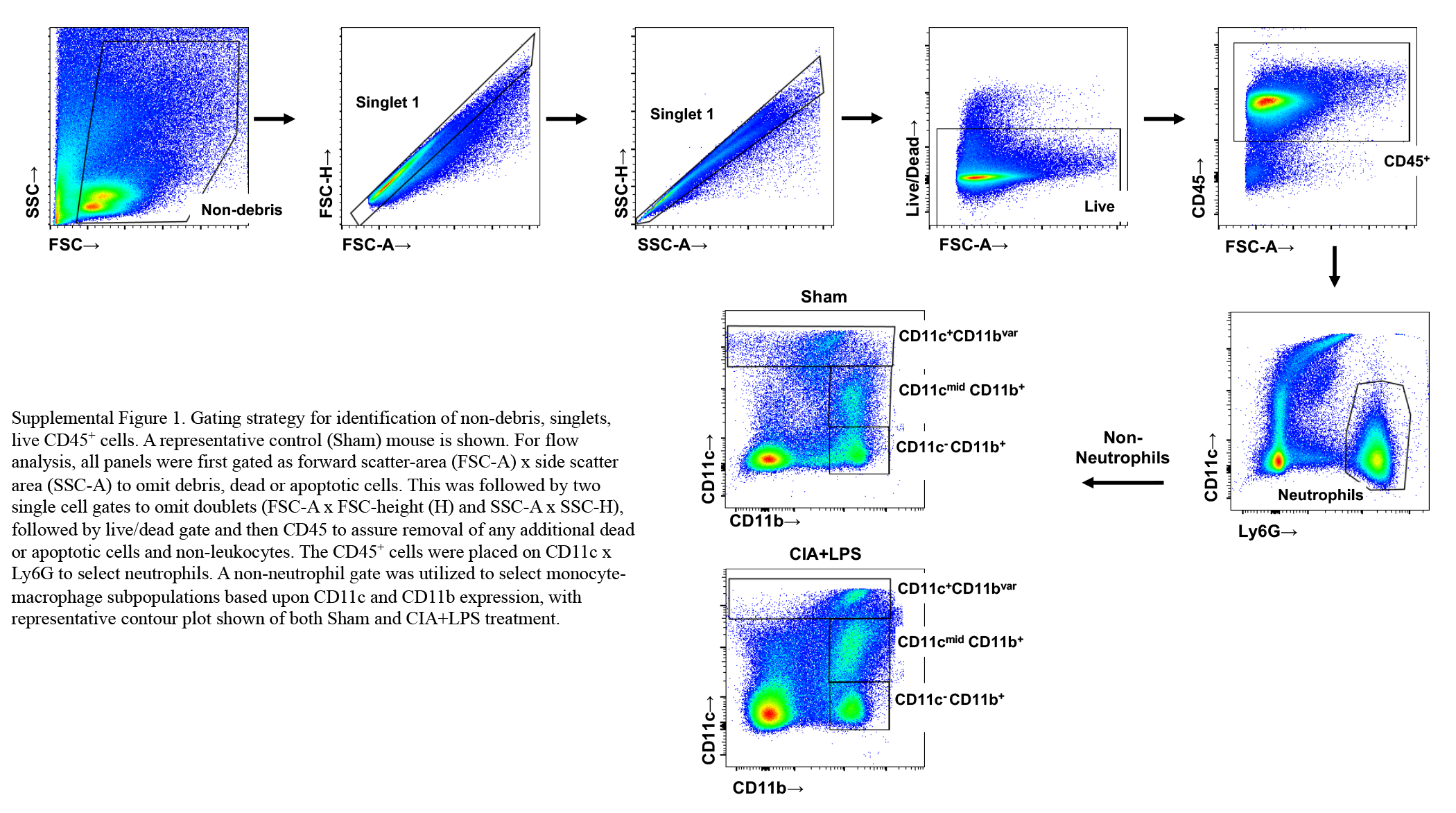

Supplement: Supplementary file 1 [file Image1.tif]

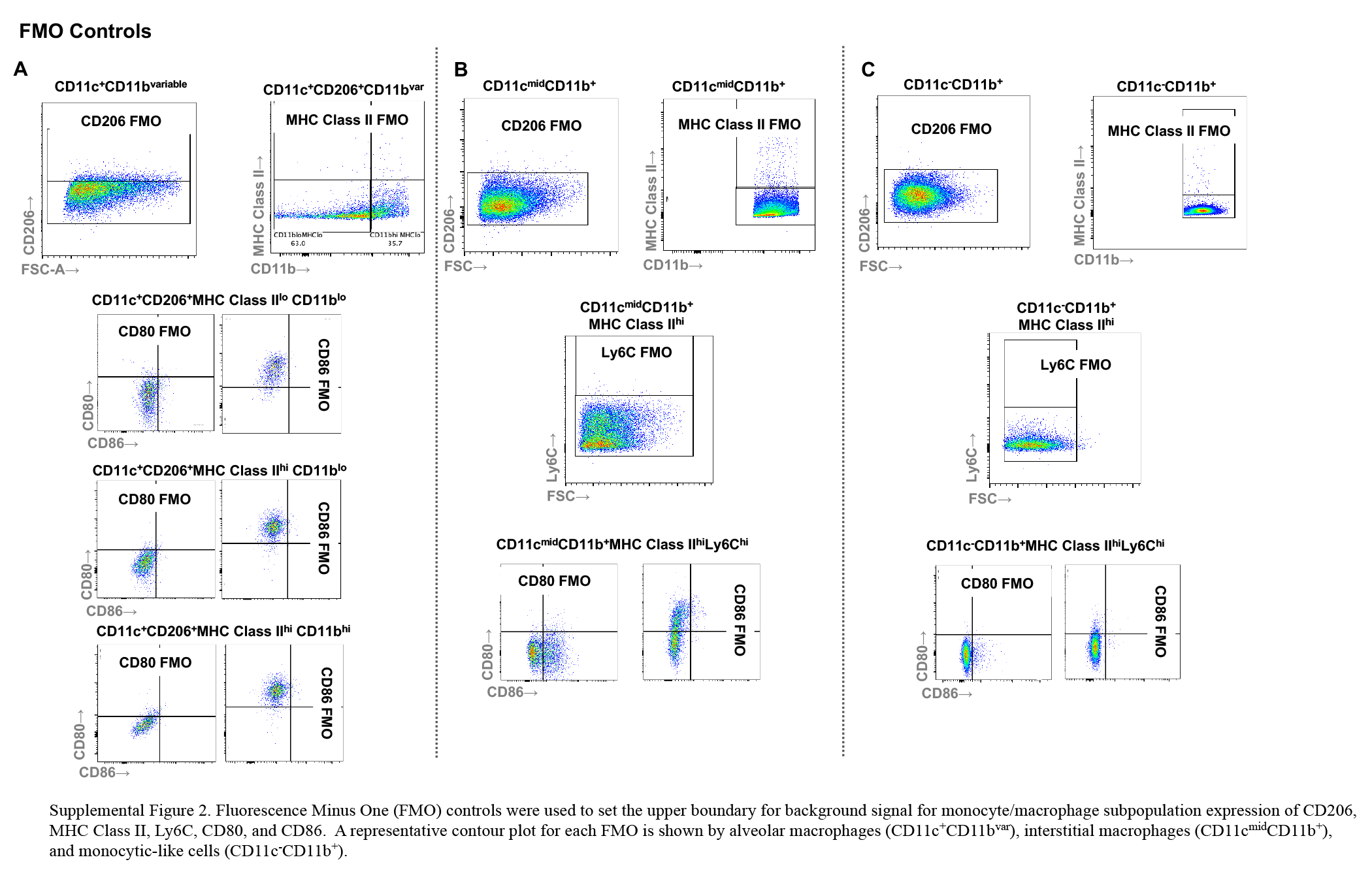

Supplement: Supplementary file 2 [file Image2.tif]

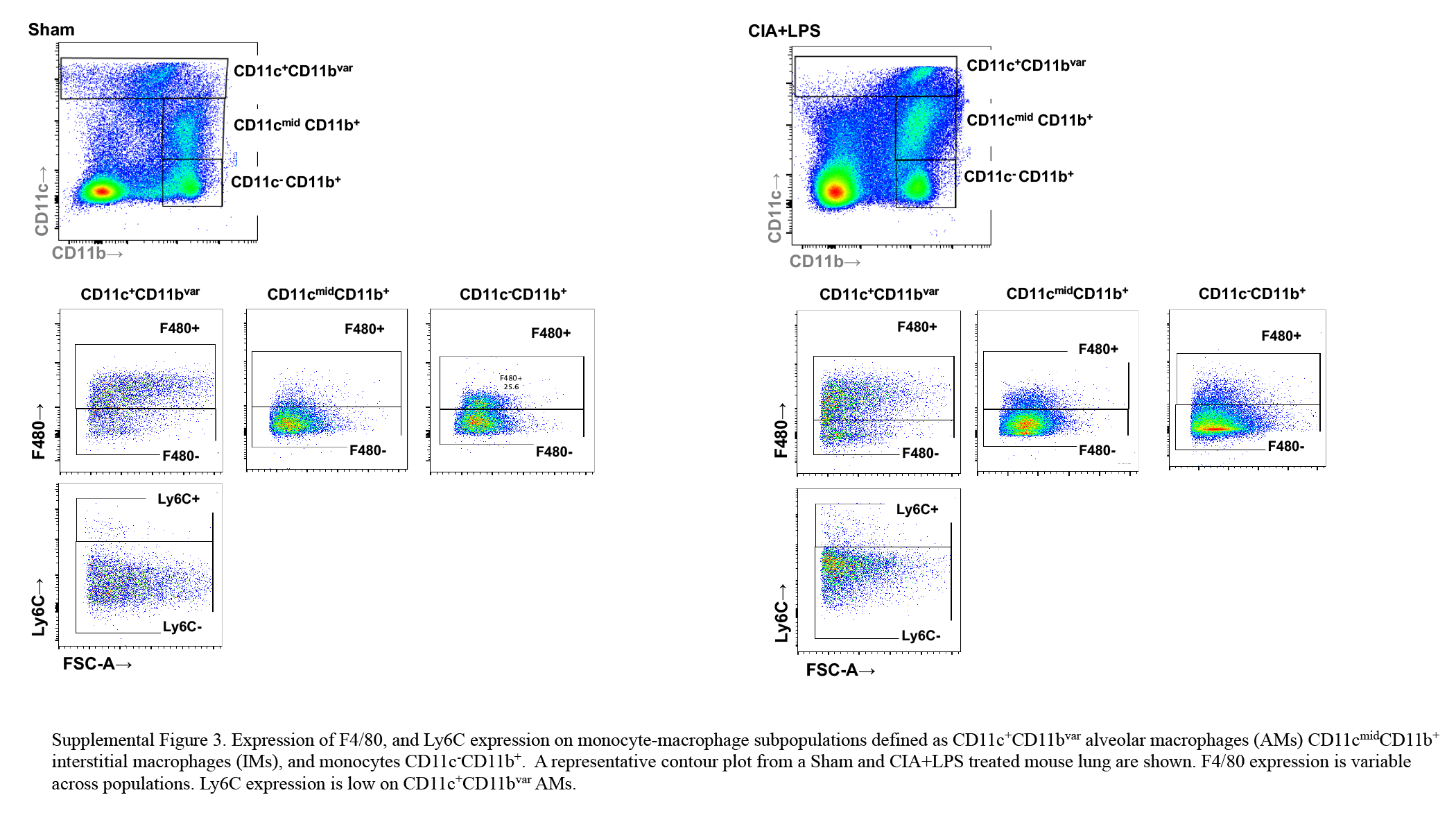

Supplement: Supplementary file 3 [file Image3.tif]
